# Are shared streets acceptable to pedestrians and drivers? Evidence from Virtual Reality experiments

**DOI:** 10.1371/journal.pone.0266591

**Published:** 2022-04-15

**Authors:** Lurong Xu, Taeho Oh, Inhi Kim, Xiaojian Hu

**Affiliations:** 1 Jiangsu Key Laboratory of Urban ITS, Southeast University, Nanjing, Jiangsu, China; 2 School of Transportation, Southeast University, Nanjing, Jiangsu, China; 3 Monash Institute of Transport Studies, Department of Civil Engineering, Monash University, Clayton, Australia; 4 Department of Urban Systems Engineering, Kongju National University, Gongju-si, Chungcheongnam-do, South Korea; 5 Jiangsu Province Collaborative Innovation Center of Modern Urban Traffic Technologies, Southeast University, Nanjing, Jiangsu, China; 6 National Demonstration Center for Experimental Road and Traffic Engineering Education, Southeast University, Nanjing, Jiangsu, China; Tongji University, CHINA

## Abstract

While the development of cities tends to focus on improving traffic mobility, it has gradually neglected people’s demand for safety and comfort walking on the streets. To address this problem, shared streets that can integrate traditional street life and traffic mobility are getting more attention as pedestrian-friendly development. In order to measure the performance of shared streets, it is essential to identify how people feel when driving and walking around. However, investigating the various factors that influence the real world is not straightforward because of cost, time-consuming, and safety problems. Virtual reality and the Human-in-the-loop (HITL) have become valuable tools for conducting experiments without compromising them. The experiments are performed on both pedestrians’ and drivers’ sides. The three shared street layouts in a virtual environment are designed according to Europe’s real shared street cases. To evaluate shared street effects, questions in five aspects: amenity, walking or driving experience, safety, economy or priority, and environmental perception are asked to participants, respectively. MPR, EWM, and Fuzzy Comprehension Evaluation methods are used to assess the performance. The result revealed that different groups of people have different sensitivity and preferences for each evaluation criteria. However, the results of the comprehensive evalutation showed that scenario C with the largest isolation measurement is preferable in both pedestrian and driver’s groups based on shared street design elements. The city planners can get help from this shared street analysis, where the new design and layout could be tested in advance.

## 1. Introduction

Previously, commercial and tourist areas were regarded as living spaces where people could hang out and enjoy their time. However, with urbanization and the increased number of vehicles, these spaces have been separated people from the vehicles to keep street users safe and traffic efficient. Even though the areas are separated by the traffic facilities to protect pedestrians from vehicles, people still feel nervous about speeding vehicles right next to the pedestrian area. Besides, people walking on the pedestrian street could get injured since speeding vehicles can penetrate traffic safety facilities and invade pedestrian zones.

In China, there are a lot of tourist and business districts that still do not take into account the accessibility and amenity of the traffic simultaneously. Many specific pedestrian lanes can improve the safety of pedestrians by restricting the entry of motor vehicles. However, they cause inconvenience and traffic congestion in the surrounding area. As an alternative way, in the Netherland of the late 1970s, there has been an attempt to slow down vehicles naturally without depriving the road users of either motor vehicles or pedestrians of their right to travel. The concept of a shared street called Woonerf, the coexistence of vehicles and pedestrians on the same street layer, was introduced. Woonerf is one of the techniques for traffic calming that yield to each other by putting cars and pedestrians into the same street layer. This method consists of three factors that help coexist with cars and people. The three factors are street environment design, traffic facility deployment, and humans. The essential element is the human being. Each person would feel different walking safety and driving environments depending on the design and location and type of transportation facilities. Therefore, the systemic shared space development analysis must investigate the effect of human factors. Besides, the development of streets in the real world exists various problems, such as construction time, large budget, and the risk of collision of pedestrians and vehicles when the improvement effect is insufficient.

In order to minimize these risk factors, this study utilizes virtual reality (VR) and Human-in-the-loop (HITL) technology. VR technology is a computer vision technology that enables people to experience the artificially created virtual world immersive. HITL technology allows human-computer interaction and fills the gap that the computer cannot implement with human factors, resulting in simulation results that consider human factors. A simulator platform that integrates the two technologies is be established from a pedestrian’s and driver’s point of view. A practical case study with the simulator checks the experiment about the different designs and strategies on shared space.

This research starts with the curiosity: 1) How does the appearance of shared street layouts affect walking and driving behavior characteristics? 2) Do aesthetic design elements have correlations to improve street safety?

This paper is divided into five parts. The second section reviews the concept and safety considerations of shared streets. In addition, VR and HITL technology are involved in this part. In the third section, the layout and design philosophy of scenario settings, the building of the virtual environment, the procedure of simulation experiment, questionnaire survey, and quantitative evaluation are described in detail. Section 4 presents the explanation and discussion of the result from the simulation experiment. The last section concludes the experiment results and proposes suggestions for the future development direction.

## 2. Literature review

### 2.1 Shared streets

Karndacharuk, Wilson [1] explained the shared space’s fundamental differences between traditional mixed streets and shared streets. In shared streets, traditional street facilities such as curbs, signs, and signals are replaced by integrated and people-oriented public spaces. This measure will encourage social interaction, walking, biking, accessibility and reduce the speed of motor vehicles. Shared streets can play a huge potential in commercial districts and residential areas. Public seating, artwork, and landscaping can significantly stimulate the vitality of urban life as an extension of the front yard and create a comfortable community life [2]. In order to make the role of shared space-efficient, deploying design elements on the shared streets is necessary [3]. From an aesthetics, acoustics, and environmental science perspective, color design elements can play an important role in calming the driving behavior on the street. Besides, coloring on the street components helps distinguish the different roles of streets, such as the safe pedestrian zone and warning of caution factors, such as tunnels and barriers [4]. Hamilton-Baillie [5] presented safer and more vibrant streets by reshaping the experience in the urban area through public arts. Biddulph [6] insisted that the rational layout of aesthetically creative transportation facilities would be able to maximize the advantages of shared streets under the premise of considering the social-cultural background.

When it comes to the safety of the shared street, some people remain skeptical about the new road format. It removes all the physical factors between pedestrian zones and streets compared to conventional street layouts [7]. As a novelty design conception, the ultimate objective of the shared street is to make the "mental speed bump" effect through the interaction of the human vehicle. The mental speed bump effect intends to ask the driver to pay more attention in situations by blurring the boundary between the street for the cars and pedestrians [8]. Nonetheless, vulnerable road users tend to feel uncomfortable and dangerous passing on the shared streets due to the absence of physical divider compared to traditional streets [9]. However, some research shows that the shared street layout is safer by encouraging yield between road users. Ruiz-Apilánez, Karimi [10] showed that shared streets are capable of preventing traffic accidents through comparison between traditional streets. Obeid, Abkarian [11] investigated the yielding behavior between drivers and pedestrians in the mixed street from the driver’s perspective using a driving simulator. The result showed that the yielding rate was higher in the shared street. The other result based on the Kruskall-Wallis method showed that driving behavior close to pedestrians was statistically less aggressive, particularly on the streets with lower speed limits.

Regarding the qualitative assessment of shared streets, Ruiz-Apilánez, Karimi [10] described the layout and performance of different shared streets in six areas from the survey. It summarized how these different spatial layouts affect street safety, amenity, and the distribution of activities to the street users. Charlton, Mackie [12] recognized that the perceptual limits of pedestrian areas vary depending on the street layout, which shows that street design is a powerful tool to change the built environment. Using the surveys and expert interviews, Karndacharuk [13] presented five different evaluation standards on shared streets, such as place, pedestrian, vehicle, economic, safety. The five criteria were used for pedestrian interaction improvement, vehicle speed reduction, and revitalizing land use for successfully shared space operations.

However, few previous studies focus on systemic analysis that can comprehensively consider various human factors. The existing literature is usually evaluated from a single perspective (drivers or pedestrians) while rarely discusses the street’s performance from two aspects simultaneously. It is essential to combine the two perceptions to get the final evaluation.

### 2.2 Virtual reality

Since virtual reality technology has become accessible, it has been widely used in various fields [14]. Lok, Ferdig [15] applied virtual reality technology to medical education. Tsai, Hsieh [16] used VR technology to orthopedic surgery simulator that illustrates the successful implementation in virtual surgery. Meggs, Greer [17] found that interior design teaching with VR technology could significantly enhance student participation and learning output. Portman, Natapov [18] recognized the use of VR in architecture and landscape.

However, Virtual Reality technology is still in a stage of continuous development, and there are undoubtedly some shortcomings in popularization. For example, Anthes, García-Hernández [19] raised the issue of the limitation of hardware devices. Since the quality of the equipment is directly related to the immersive experience, much financial support is required. Besides, improper VR devices negatively affect users, such as dizziness and headaches [20] because of the lag between screen imaging, the gap between the 3D and the actual image, and the discomfort of wearing a helmet. Therefore, VR technology is unsuitable for all conditions at the application level of education and training, especially for older teachers whose teaching methods are challenging to change. If they cannot adapt well to the difference between VR technology and traditional teaching methods, it is difficult for them to play a positive role in VR teaching [21].

Although immature technology development limits the application of VR technology, it has enormous potential in traffic simulation. Meir, Oron-Gilad [22] simulated the crossing scenario and let the child participate in the virtual environment experiment. Through the risk assessment test, he tested the risk perception ability of children in this traffic environment from the perspective of pedestrians. Chun, Ge [23] proposed a method to use VR technology to convey the design plan to city planners, verifying the significant role that VR technology can play in transportation planning. In addition, the immersion of VR technology is unmatched by pictures and text information. In response to the ethical dilemma of autonomous driving, Sütfeld, Ehinger [24] used VR technology and text-based surveys to test the decisions made by different participants in the face of different factors. The entire experiment further verifies the application of VR technology in autonomous driving.

The above research confirmed the immersion and interactivity of the VR technology and demonstrated its potential in realizing realistic surrounding environments for the experiment.

### 2.3 Human-in-the-loop

Human-in-the-loop (HITL) with VR is defined as a model that plays a vital role in the simulation due to human-computer interaction in real-time impacts on research results [25]. The HITL has been widely used in the evaluation of industry design and the exploration of environmental science. Kefalidou, D’Cruz [26] focused on the degree of comfort. User-centered airport interior design through 3D visualization and emotional test for passengers verified the value of HITL with VR in practice and operation. Jiang, Masullo [27] experimented using VR through simulating street-design scenarios to explore the influence of soundscape from a human perspective.

In the field of traffic engineering, there is plenty of research that proves the applicability of HITL. In order to investigate traffic safety from a pedestrian’s perspective, Deb, Carruth [28] developed a pedestrian simulator to investigate the safety issue in signal intersections. Lei, Yalian [29] established a driving simulator with the Unity 3D game engine to test the fuel consumption of hybrid electric vehicles. Hartmann, Viehweger [30] examined the anti-collision system for autonomous vehicles to protect the pedestrian’s safety. Oh [31] proposed an emerging platform named Human in Virtual Reality in the Loop Simulator (Hi-VRiLS) and verified that human factors need to be considered in safety analysis simulation.

In the field of transportation, HITL technology is widely used in traffic safety research. However, there are occasional papers considering human factors by this technology. Moreover, the shared streets research corporation with VR technology is still missing. Therefore, shared street research based on the HITL model and VR technology is meant to look into the human interaction in simulation.

## 3. Methodology

**Fig 1:**
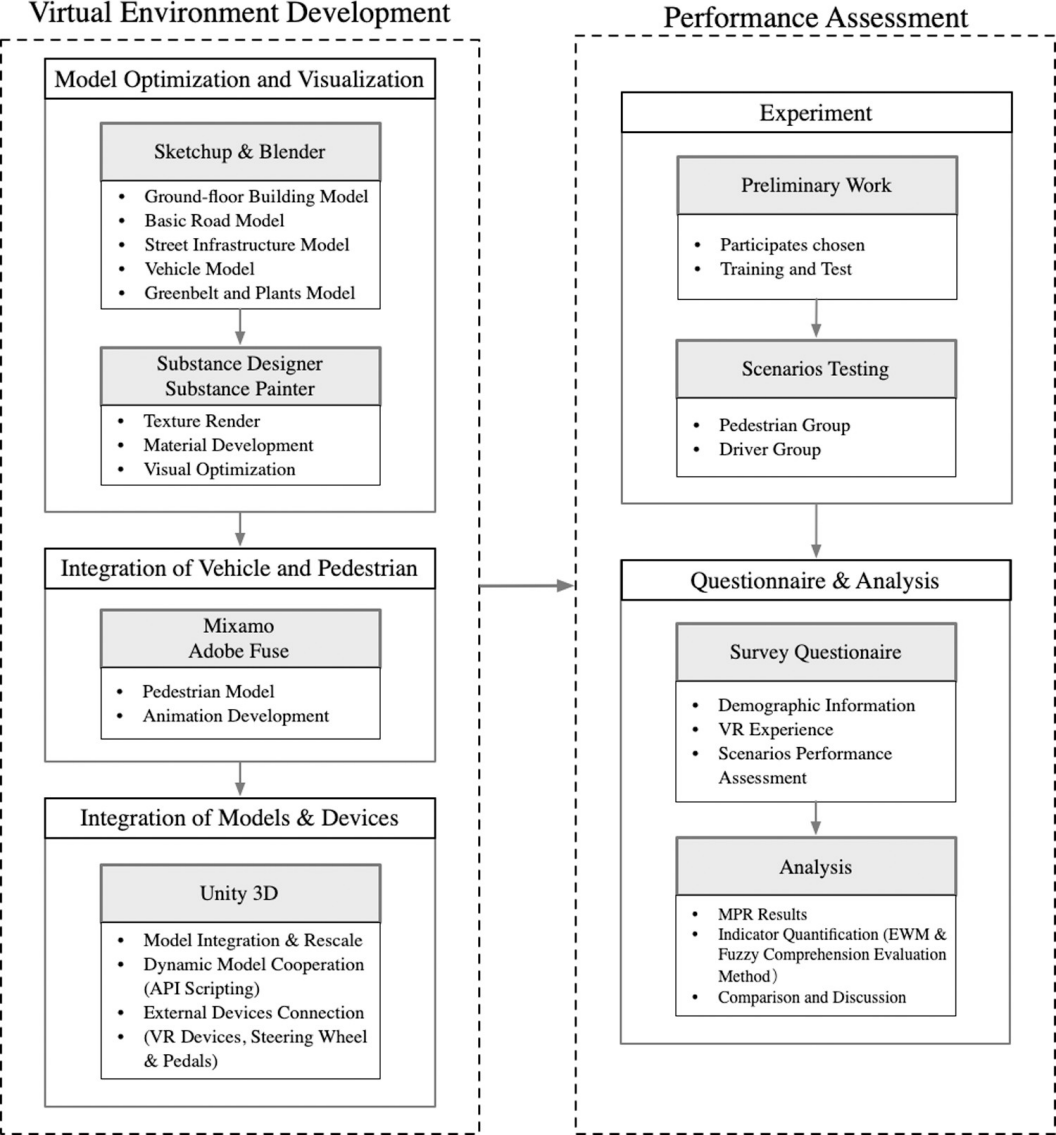
Framework of experiment development.

This research is divided into two sections, as shown in Fig 1. The first section is to build a virtual experiment environment of shared streets according to traffic facilities’ different designs and deployment. Three scenarios are selected for the analysis based on the experience of shared space projects in various countries. The second section assesses the user reactions depending on roles in the differently built shared street environment.

In the first step, the components such as background environments, pedestrians, and vehicles were built. Sketchup and Blender are utilized to establish the experiment background environments. Building and streets built in Sketchup are imported into Unity3D, and Blender is used to adjust Sketchup and Unity3D if there is a rendering error. After that, Substance Designer and Painter tools are used to handle the texture and materials on the building walls and street components for better realistic scenery. In order to create the pedestrian models, Adobe Fuse is used for creating the basic human appearance and outfits to Mixamo for allocating the movement to each pedestrian model. Lastly, all the buildings and pedestrian models are imported in Unity 3D to have an actual street environment. For the driving experience, VR devices and driving equipment, such as head-mounted devices, steering wheel, and pedals, are included. A complete traffic system is also implemented using scripting, in which pedestrians and vehicles can interact and yield to each other in a virtual scene. This script mimics the reality of simulation to the greatest extent and can improve the accuracy of the experimental results.

In the second step, an experiment and questionnaire survey about the experience of shared streets are conducted for a performance assessment from both pedestrian’s and the driver’s point of view. A total of thirty-two volunteers joined in this experiment and were divided into two groups. After finishing the experiments, all the participants are asked to share the streets experience three scenarios and VR device experience.

Based on the collected data from the questionnaire, the Median Perception Rating (MPR) measure is adopted to evaluate the performance of three scenarios following different criteria. Entropy Weight Method (EWM) and Fuzzy Comprehension Evaluation methods integrate all the criteria for making a comprehensive assessment.

### 3.1 Study area selection

For the shared street experiment, a segment of straight street where the Suzhou Museum is located was selected as the study area. The street segment is approximately 832 meters long and 10 meters wide. There are various shops on both sides of the street, coupled with the attraction of tourist destinations, the traffic on this street is huge. However, the road condition around the Suzhou Museum is lagged. Therefore, the street is often suffering to take the huge tourism demand causing the traffic congestion and dangerous conflict occasion between pedestrians and vehicles happens frequently. In order to address traffic congestion, this street was transformed into a specific pedestrian section in 2018. This transformation has caused inconvenience to the residents that vehicles have to make a big detour to avoid this area. Therefore, it is a suitable segment as a case study to investigate the effectiveness of shared streets. Simultaneously, it is good to combine mobility and walkability and stimulate livability and commercial development. Three different street layouts are established based on different attributes (see Table 1).

**Table 1:** The attributes classification of scenario settings.

Attribute		Scenario A	Scenario B	Scenario C
**Isolation**		None	Soft	Strong
**Pavement**				
a)	Paved Guidance	None	Curved Red Line	Straight Red Line
b)	Surface Material	None	Adjust rough material on red marked road	Adjust rough material on red marked road
**Parking Area**		None	None	Along the Grey Pavement
**Aesthetic Elements**				
a)	Trees or Plants	Yes	Yes	Yes
b)	Seating Facilities	None	Deploy pavilions and promenades	Deploy pavilions and promenades

#### 3.1.1 Scenario A

**Fig 2:**
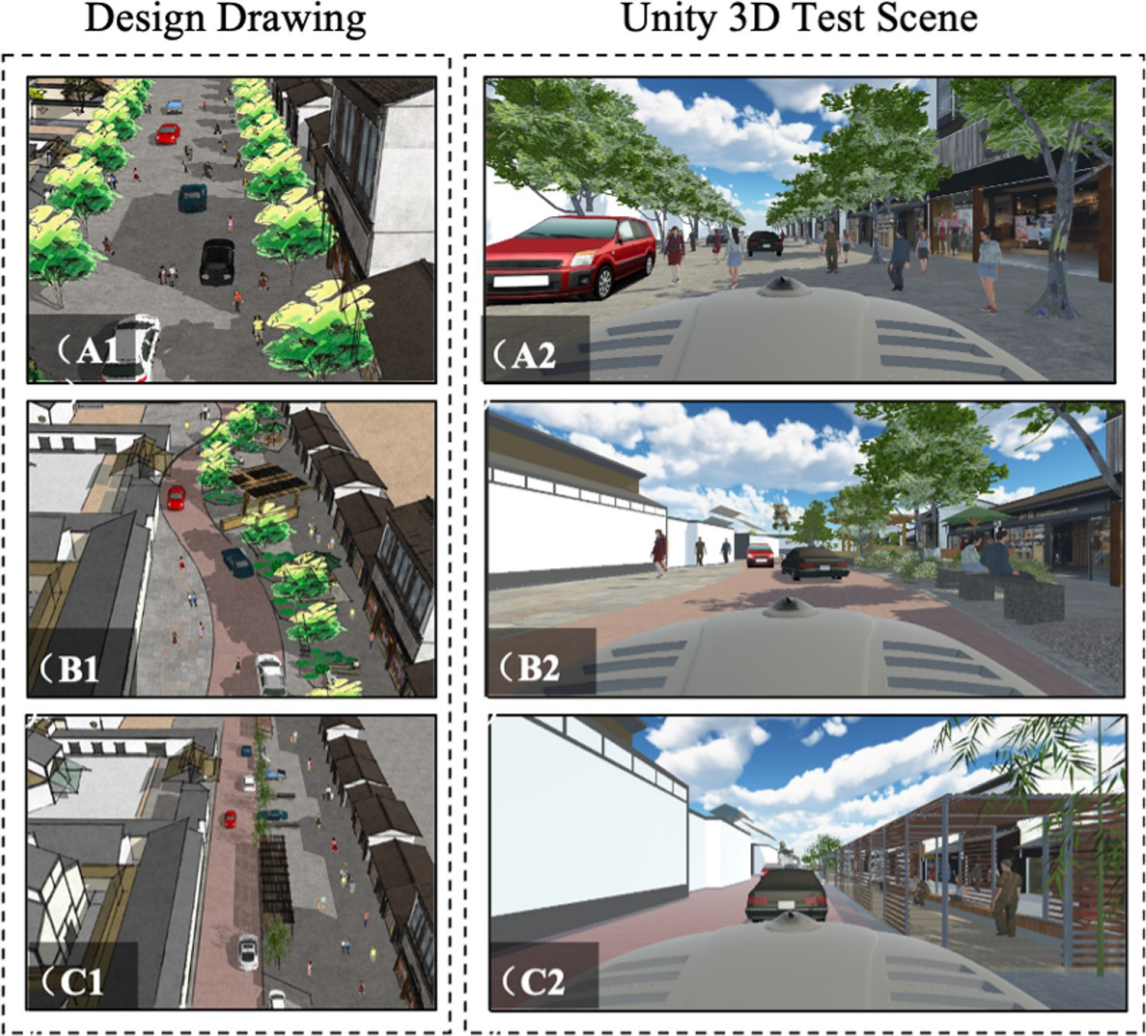
Scenarios setting.

According to the concept of Woonerf, scenario A is a shared space with a homogeneous pavement. Pedestrians and vehicles can freely move around (Fig 2A). This scenario aims to strengthen the "mental speed bump" effect that encourages human-vehicle interaction by removing facilities that split vehicles and pedestrians on the street. This concept intends that pedestrians freely crossing the street bring uncertain environmental prompts to the driver. By giving pedestrians more freedom on the streets, the majority of users in street spaces can be transferred from vehicles to pedestrians. Pedestrians are expected to get more guaranteed priority to travel the whole area [32].

In addition, the unified and continuous street pavement can change the overall atmosphere of the space and provide people with places to stay and play. The uniform paving can also improve comfort for pedestrians to increase attractiveness so that pedestrians gradually become the protagonists of the street user while vehicles become intruders. By doing this, drivers put more attention on the streets while driving to avoid accidents with people in shared spaces that can happen to various activities.

#### 3.1.2 Scenario B

Following the space reconstruction case of Garibaldi-Brera Environment Island [33], scenario B includes traffic calming factors combined with the traditional Woonerf concept (Fig 2B). In addition, the aim of traffic safety measures has shifted from restricting speed and flow to creating harmony between people and vehicles by making shared street dynamics [34]. Therefore, scenario B intends to ask drivers to drive slowly unintentionally. People are more likely to behave more cautiously when exposed to complex, attractive, and uncertain environments for harmony between drivers and pedestrians.

When it comes to the pavement, the red paving material is much rougher than the gray-colored road material. By doing this, vehicles on the red road slow down naturally since they get the frictional force. In addition, the driving path guides the cars to drive the S-shape of the street, reducing the driving speed dramatically.

Facilities and environmental elements on the streets enrich the scene and enhance the psychological satisfaction of users. The trees were deployed to create the effect that trees delimit a pedestrian zone. Facilities were also placed to make people enjoy their time on the streets, such as tables, chairs, and stores.

#### 3.1.3 Scenario C

The pedestrian zone is placed parallel to the straight roadway. It is separated by plants and facilities based on a design of the South Kensington [10] (Fig 2C). In addition, there are spaces for parallel parking along the street. Several public facilities for pedestrians are located to make people stay longer on the street. The advantage of this is increasing the sense of walking environment safety by dominating the area and increasing the number of walking activities [35].

Compared to the previous two scenarios, the most considerable difference was that scenario C separated the role of the street using facilities and different pavement with the same driving way.

### 3.2 Virtual environment development

#### 3.2.1 Experiment environment establishment

Several applications were used to make a virtual experiment environment consisting of static and dynamic models. When it comes to static models, the structure of base models was created using SketchUp, which allows making buildings and street segments. Once the shared street layout sketch was done, Blender, an open-source modeling software, was used for model optimization since it has powerful rendering functions. Besides, given the ability to interact with other visual modification software, Substance Design and Substance Painter was able to cooperate with Blender as well as Unity 3D successfully. Furthermore, the entire workflow referenced from a game development remarkably achieved visual fidelity. It conformed to the characteristics of aesthetic development.

**Fig 3:**
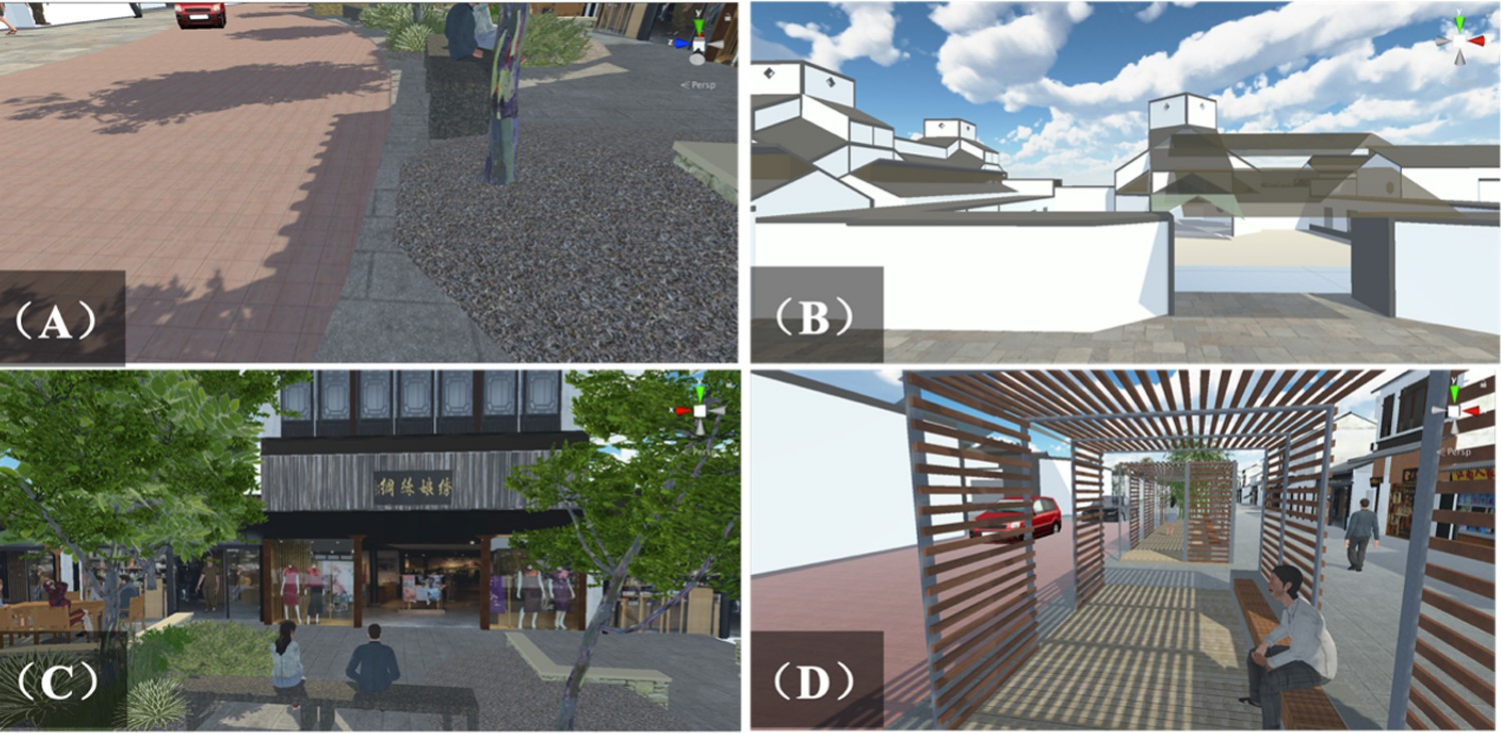
Visualization in unity 3D.

Fig 3 shows the detailed visualization created by the workflow. Considering the texture of the paving material had an important influence on the realization of the concept of street layout in this research, Substance Painter was used to achieve visual authenticity and aesthetics. Simultaneously, the substance designer focused on material restoration when driving on the street. The different materials could convey distinct feedback to the participants through the steering wheel (see Fig 3A). Moreover, high-definition textures were used to achieve a realistic environment (see Fig 3B Suzhou Museum, Fig 3C Commercial Stores, and Fig 3D Street Furnitures, respectively).

#### 3.2.2 Street users setting

In order to make a realistic street environment, the people and cars were controlled by computer programming from the asset package in Unity 3D called Urban traffic system 2018. They randomly walked and drove around naturally. Performing the high similarity to the Exhibition Street-Museum mentioned in [10], traffic volume and speed were 600veh/h and 30 km/h, which is the actual speed limit in the area. Besides, the volume and walking speed for the pedestrians were 300ped/h and 5 km/h, respectively. It is noteworthy that when the distance between two street users is less than two meters, they yield to each other.

Moreover, Mixamo was used to give animation to the fixed pedestrian model. The people in the virtual world behaved more realistically by walking, laughing, talking, and sitting.

### 3.3 Experiment

This experiment was carried out in Suzhou, China, in January 2020. All the participants were postgraduate students at Southeast university-Monash university Joint Graduate school. They were recruited by an advertisement posted on social media (e.g., Wechat group) with the restriction of having a driver’s license for at least more than a year. This experiment was approved and authorized by Southeast University. We also introduced the voluntary statement on the first page of the survey questionnaire, which includes the related terms of Withdrawal (During the process, participants can withdraw from the investigation at any stage at any time) and Confidentiality (Only the thoughts and opinions volunteered by the participants in the survey will identified in the data collection, and none of them will be identified by name. The findings will be used for publication purposes in the form of papers, journal articles or conferences). The survey is completely CONFIDENTIAL, ANONYMOUS, and VOLUNTARY. The detailed process of integrating Unity 3D and VR equipment is described in [31]. After choosing participants, this experiment can be divided into five steps: introduction, grouping, testing, scenario experiment, and questionnaire survey.

In the first step, the concept of shared streets was briefly introduced to the participants. Then, all participants were divided into two groups with equal sample sizes randomly. The participants in group one experimented with a driver using driving devices, including the steering wheel and pedals. The participants in group two played a pedestrian role using gaming controllers. In the third step, all the participants were required to adopt the VR environment and experimental equipment in a test scenario until being familiar with the VR environment and controlled before the actual experiment. In step four, each participant was required to experiment with three scenarios. Participants got three minutes of break between the scenarios. During the experiment, each participant in the driver group took an average of fifteen minutes, while participating in the pedestrian group took thirty minutes. In the end, all participants were required to answer the questionnaire. In this experiment, 34 respondents were recruited. Among them, two participants wanted to quit the experiment due to dizziness. Therefore, a total of 32 valid questionnaires were collected and recorded.

**Fig 4:**
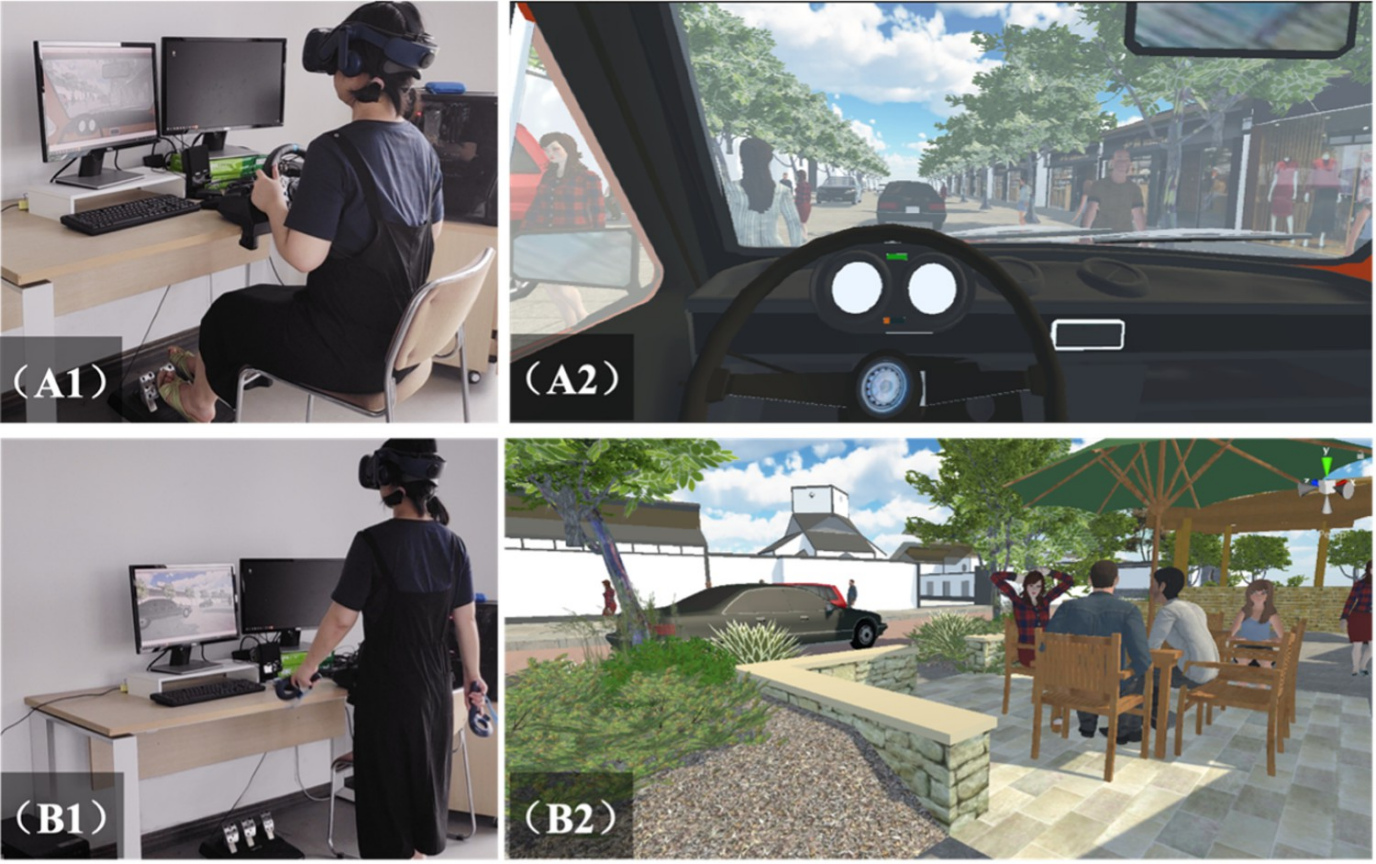
Experiment environment.

Fig 4 shows the simulation environment. Specifically, A1 and A2 show the environment in reality and scene in Unity 3D of the driver group, and B1 and B2 show the environment in reality and scene in Unity 3D of the pedestrian group.

### 3.4 Questionnaire survey

In order to conduct a performance assessment, pedestrian satisfaction factors were considered, such as ’Pedestrian Environment Review System (PERS)’ and ’Pedestrian Environment Data Scan’ [36, 37]. Based on the 6-point Likert rating scale proposed by Bradburn, Sudman [38], the evaluation criteria range is from ’-3’ to ’+3’, representing ’strongly disagree’ and ’strongly agree’. This scoring method deliberately removes the neutral "0" point so that participants can be more cautious when scoring. It makes results more reliable and intuitively reflects positive and negative evaluations. In addition, the level of satisfaction of shared streets was evaluated in five different aspects following Karndacharuk [13].

During the experiment, a pilot survey was conducted among 20 randomly selected respondents. The questionnaire was revised and ensured accuracy according to the feedback of these respondents. The questions are divided into two types: group and common questionnaire (Table 2). Participants in the different groups were asked to answer to their shared space experiences according to their roles. The group questionnaires included five criteria, such as comfort, freedom, safety, habits, and environment. After that, the common questionnaire to know awareness of shared streets and VR experience were asked.

**Table 2:** Design of questionnaire survey.

Group Questionnaire (scale from -3 to +3)		
Criteria	Pedestrian Group	Driver Group
Comfort	I felt comfortable walking	I felt comfortable driving
Walking/Driving Experience	I could freely move around	I could drive smoothly
Safety	I felt safe and secure in the street.	I would like to drive slowly
Economy/Priority	I enjoyed time in the street	I preferred to yield to pedestrians.
Environment	I felt the traffic noise is reduced compared to conventional streets.	
**Common Questionnaire**		
Have you ever heard of the shared street before? (a)Yes, (b)No.		
Have you ever experienced VR before? (a)Yes, (b)No.		
Do you ever feel dizzy using the VR driving simulator? (a)Yes, (b)No.		
Your opinion of shared street’s layout compared to conventional streets.		
**Demographic Information Questionnaire**		
What is your age?		
What is your gender?		

### 3.5 Quantitative evaluation method

For the evaluation, Median Perception Rating (MPR) proposed by Torgerson [39] is used as a qualitative indicator to analyze the performance. The same qualitative assessment method is leveraged by Karndacharuk, Vasisht [40] in the before and after transformation case analysis of shared streets.

The following steps can conclude the detailed process of the MPR method:

Step 1: Organize the data collected from the questionnaire survey and calculate the proportion of respondents in each score rate.
Step 2: Conduct a cumulative calculation following the response proportion; each score rate is the upper boundary.
Step 3: Plot the cumulative curve in the calculation results in step 2. The abscissa is the score rating from "-3" to "+3" and the ordinate is the proportion value from 0 to 1.
Step 4: By determining the point of the curve at 0.5 on the ordinate, find the value corresponding to the abscissa and obtain the MPR value.

The Entropy Weight Method (EWM) method is an objective weighting method that can be used to evaluate the influence of a particular index on a comprehensive evaluation. The following formula presents the procedure to conduct the EWM method:

Step 1: Define the number of samples as n, number of criteria as m, use **Eq 1** to conduct normalization. Where, *x*_*ij*_ represents the value of sample *i* for criteria j(i=1,…,n;j=1,…,m).

$$v_{ij}=\left\{ \begin{array}{c} \frac{x_{ij}-min(x_{j})}{max(x_{j})-min(x_{j})}\\ \frac{max(x_{j})-x_{ij}}{max(x_{j})-min(x_{j})} \end{array}\right.$$ (1)

Step 2: determine the eigenvalue proportion of sample *i* under the evaluation criteria *j* using **Eq 2**:

$$p_{ij}=\frac{v_{ij}}{\sum_{i=1}^{n}v_{ij}}$$ (2)

Step 3: the calculation of information entropy of sample *i* under the evaluation criteria *j* using **Eq 3**:

$$e_{ij}=-\frac{1}{ln(n)}\sum_{i=1}^{n}p_{ij}ln(p_{ij})$$ (3)

Step 4: Define the diversity factor using **Eq 4**:

$$d_{j}=1-e_{j}$$ (4)

Step 5: Calculate the weight of each criteria using **Eq 5**:

$$w_{j}=\frac{d_{j}}{\sum_{k=1}^{m}d_{j}}$$ (5)

The fuzzy Comprehension Evaluation method can transform qualitative evaluation into quantitative evaluation based on the membership theory.

Step 1: Define the number of samples as n, number of criteria as m. Establish a comprehensive evaluation criterion set *U* = (*u*_1_, *u*_2_,⋯,*u*_*m*_), and a comprehensive evaluation grade set *V* = (*v*_1_, *v*_2_,⋯,*v*_*m*_).
Step 2: Build a fuzzy relationship matrix using **Eq 6**:

$$R=[\begin{array}{c} R|u_{1}\\ R|u_{2}\\ \cdots \\ R|u_{m} \end{array}]=[\begin{array}{cccc} r_{11} & r_{12} & \cdots & r_{1n}\\ r_{21} & r_{22} & \cdots & r_{2n}\\ \cdots & \cdots & \cdots & \cdots \\ r_{m1} & r_{m2} & \cdots & r_{mn} \end{array}]$$ (6)

Step 3: Determine the weight vector of criteria by AHP or EWM method and represent it as *A* = (*a*_1_, *a*_2_,⋯,*a*_*m*_).
Step 4: Build a comprehensive evaluation model using **Eq 7**:

$$A\circ R=(a_{1},a_{2},\cdots ,a_{m})\left[\begin{array}{cccc} r_{11} & r_{12} & \cdots & r_{1n}\\ r_{21} & r_{22} & \cdots & r_{2n}\\ \cdots & \cdots & \cdots & \cdots \\ r_{m1} & r_{m2} & \cdots & r_{mn} \end{array}\right]=(b_{1},b_{2},\cdots ,b_{n})$$ (7)

## 4. Performance analysis

### 4.1 Demographic characteristics

They are postgraduate students at Southeast university-Monash university Joint Graduate school in Suzhou, China. The first participants joined the pedestrian scenario (Mean age 22.16 years, SD = 1.34, 43.75% males, 56.25% females). The second group joined the scenario being drivers (mean age 22.97 years, SD = 1.16, 68.75% males, 31.25% females).

Table 3 shows the basic demographic information of participants, in which people who had not experienced the VR environment before this study accounted for the majority. However, only a tiny proportion of people reacted with dizziness during the experiment (12.50% for the pedestrian group and 18.75% for the driver group, respectively).

**Table 3:** Demographic information.

Items		Experimental Group (%)	
		Pedestrian	Driver
**Gender**			
a)	Male	7 (43.75)	11 (68.75)
b)	Female	9 (56.25)	5 (31.25)
**Age (Mean Value)**		22.16	22.97
**V.R. Experience**			
a)	Have not Experienced Before	10 (62.50)	9 (56.25)
b)	Have Experienced Before	6 (37.50)	7 (43.75)
c)	Dizzy	2 (12.50)	3 (18.75)
d)	Not Dizzy	14 (87.50)	13 (81.25)

### 4.2 Statistical analysis

Before performing the formal statistical analysis, Cronbach’s alpha test was conducted to examine the reliability of this questionnaire. The result shows that the scale is acceptable (α = 0.86), which means that the survey result is reliable and acceptable. Two ANOVAs (Analysis of Variance) were conducted to test the significance of street design impact on five shared street ratings. The results show that the street design effect of three scenarios on the ranking is significant at 0.05 confidence level in both experiment groups (df = 2, F = 5.9175, p = 0.0031 for pedestrian group and df = 2, F = 8.5825, p = 0.0002 for the driving group, respectively).

**Fig 5:**
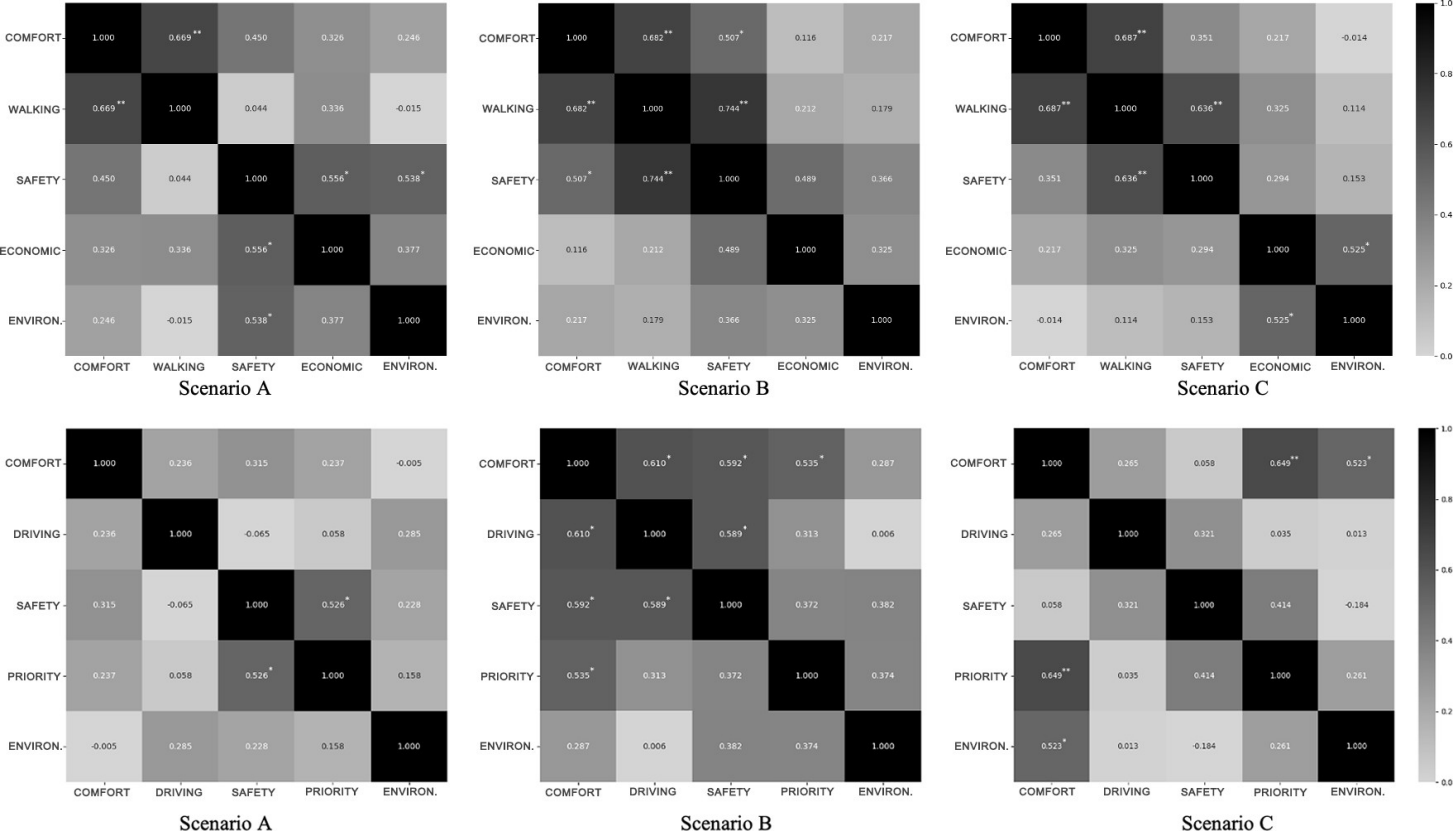
Spearman’s rank correlation matrix of pedestrian (a) and driver (b) experiment groups.

Spearman’s rank correlation matrix of two groups is presented in Fig 5, respectively. Regarding the pedestrian experiment group, comfort criteria are highly related to walking experience in three scenarios. In addition, "safety" criteria are also affected by other criteria, and this interrelationship varies in different scenarios. It is worth noting that the correlation coefficient between "safety" and "walking experience" is the highest value in the pedestrian experiment group (0.744). In contrast, the correlation relationship among the five criteria is different in the driving group. The highest coefficient value appears between "comfort" and "priority" in scenario C (0.649).

Moreover, a complex interrelationship appears in scenario B that four pairs of criteria are statistically correlated. Comparing these two experiment groups, the correlation among various criteria is evident in scenario B. Except for scenario C in the driving experiment group, at least one criterion is related to "safety".

### 4.3 Quantitative evaluation

**Fig 6:**
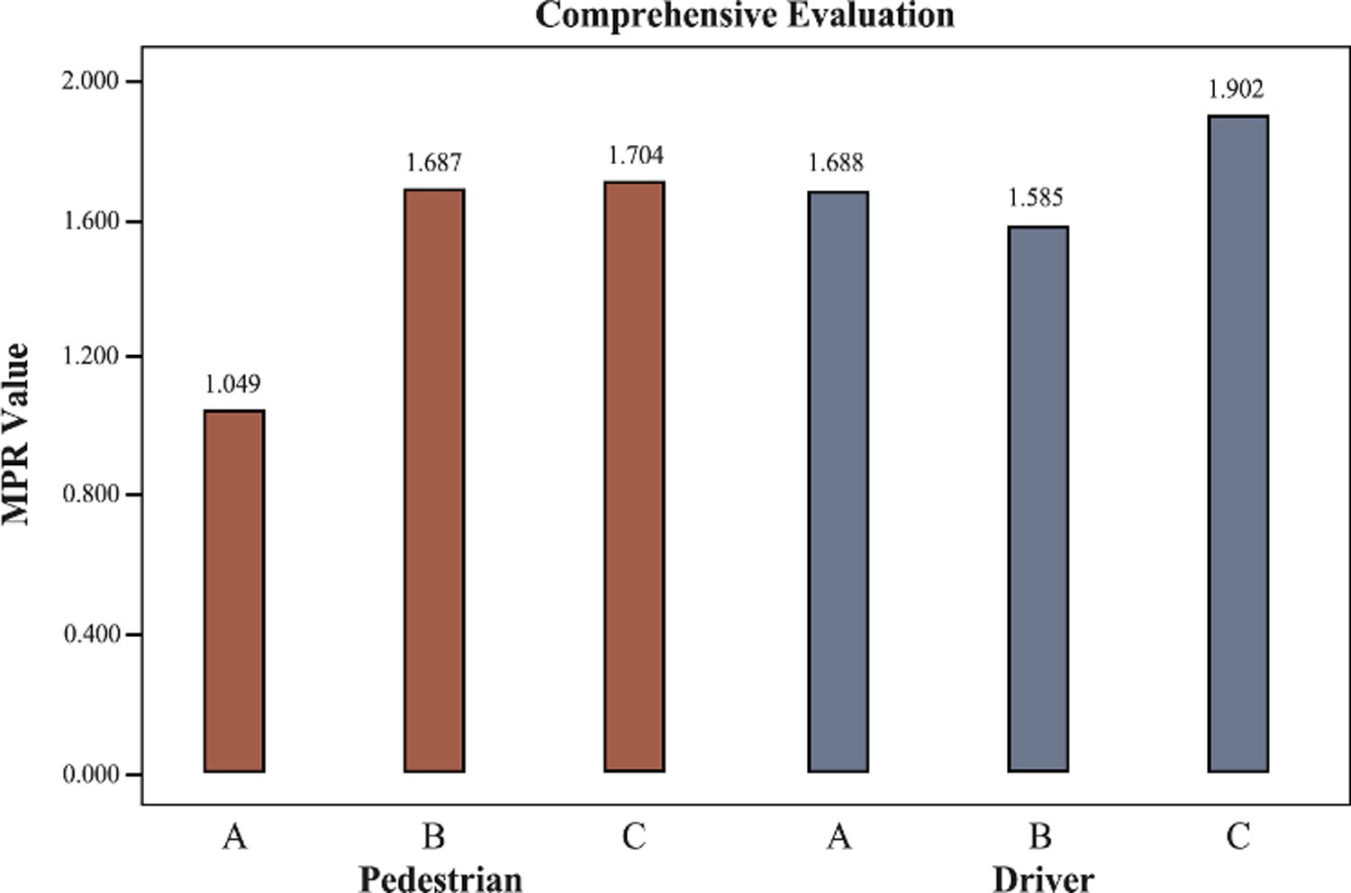
Comprehensive evaluation result.

Table 4 summarizes MPR, mean value, and standard deviation from a pedestrian and driver perspective. Plus, the Entropy Weight Method (EWM) and Fuzzy Comprehension Evaluation method are conducted the five criteria integration to evaluate three scenarios quantitatively (Fig 6).

**Table 4:** Median Perception Rating (MPR).

Index	Scenarios	Pedestrians			Drivers		
		MPR	Mean	SD	MPR	Mean	SD
Comfort	A	1.297	1.188	1.509	1.167	0.938	1.853
	B	**1.444**	1.688	1.364	1.286	1.563	1.171
	C	1.429	1.625	1.158	1.455	1.938	0.556
Walking/Driving Experience	A	1.375	1.438	1.456	0.833	0.875	1.495
	B	**1.569**	1.813	1.136	1.286	1.125	1.798
	C	1.556	1.938	1.938	1.778	2.313	0.583
Safety	A	1.063	1.000	1.248	1.222	1.250	1.346
	B	1.333	0.916	0.916	1.714	1.938	1.248
	C	**1.571**	1.938	1.029	1.375	1.750	0.968
Economic/Priority	A	0.667	0.813	1.285	1.143	0.875	1.798
	B	1.142	1.438	1.116	1.500	1.438	1.694
	C	**1.250**	1.063	1.749	1.429	1.563	1.368
Environment	A	1.143	0.750	1.953	1.125	1.062	1.435
	B	1.556	1.813	1.184	1.489	1.836	1.014
	C	**1.625**	1.875	1.218	1.556	1.938	0.966

#### 4.3.1 Pedestrian group

Scenario B was better than the other two scenarios in the comfort and pedestrian criteria performance. In comparison, scenario C was the best in the performance of the following three criteria. It is noteworthy that the MPR value of scenario A remained the lowest among the whole five criteria. With regard to mean value, even though the performance of safety perception of scenario A represented a higher value than scenario B, the other criteria show that the evaluation of scenario A was the lowest on the mean value. From the comprehensive evaluation result, scenario C had the highest pedestrian perception evaluation among the three settings.

#### 4.3.2 Driver group

It is almost identical to the MPR value of scenario A distributed among the five criteria. Different from the result of the pedestrian experiment, the MPR results reveal that scenario C outperformed the other two scenarios in comfort, driver, and environmental aspects. In addition, scenario B had a higher score in the safety and priority criteria assessment. From the perspective of the comprehensive evaluation indicator, scenario C was almost identical to scenario B. Regarding the comparison of the pedestrian group, only the performance evaluation of scenario B from drivers’ perception outperformed over the other two settings.

### 4.4 Discussion

#### 4.4.1 Recognition of shared streets

Although the aim of the street design is the harmonious coexistence of vehicles and pedestrians on the street, it might be possible that the people are aware of the shared street. To be specific, there was little difference in the MPR index between scenario B and C in the amenity level from the pedestrian group experiment. In contrast, there were huge differences between scenarios A and B as well as scenarios A and C (0.147 and 0.132, respectively). In the driver group, the MPR and Mean value of scenario A in driving experiment criteria was significantly lower than the other two settings (MPR 1.167, Mean 0.938 for scenario A, MPR 1.286, Mean 1.563, and MPR1.455, Mean 1.938 for scenario B and C, respectively). Moreover, in common questions, the opinions in scenario A showed different responses compared to other scenarios. The comments like ’chaotic’ and ’disordered’ on scenario A appear on the driver group’s questionnaire survey. It can be inferred that from drivers’ perception, completely removing all isolation measures will reduce their sensory satisfaction. However, according to the responses, the majority were optimistic about all three scenarios’ shared street layout. The popular comments were, "I prefer this kind of street compared to conventional streetways". Therefore, the optimistic opinion of the shared street is revealed.

#### 4.4.2 The difference between pedestrian and driver groups

It is noteworthy that there was an enormous difference between the two groups regarding the MPR and means value. The MPR value presented in Table 4 reveals that the drivers’ ratings for these three scenarios were generally lower than pedestrians for the criteria of comfort, driving experience, and environment. For example, for evaluating walking and driving experience criteria in scenario A, there was a massive difference in MPR value between driving and pedestrian experiment (MPR 1.375 for the pedestrian group and MPR 0.833 for the driver group). Besides, according to the comprehensive evaluation presented in Fig 6, drivers prefer scenario A more than scenario B. In contrast, there was an opposite situation in the pedestrian group. Considering the new design tends to prioritize pedestrians as "vulnerable groups", the restriction to motor vehicles has increased.

#### 4.4.3 Interrelationship among different criteria and the influence of aesthetic elements

The results of Spearman’s test present that improving one criterion is likely to improve street users’ evaluation of other criteria. For example, scenario C with the highest safety MPR score in pedestrian group also has a relatively higher performance in walking experience, which corresponds to the correlation between "safety" and "walking experience" criteria in the Spearman matrix. Much literature proposes that shared streets can effectively ensure traffic safety [5, 41]. The reason is that this kind of street layout can improve users’ comfort while removing the hard isolation, improving the traffic environment, and creating a mental speed bump for users. By exploiting the correlation among different criteria of street performance, speeding caution can be given to both drivers and pedestrians.

Moreover, aesthetic elements significantly affect performance rank, considering its importance in improving comfort, walking/ driving experience, and environment. For example, in scenario B, the change of pavement types, curved street design, and landscape location help drivers drive carefully while increasing the sense of pleasure. Concerning the higher MPR value of safety, priority, and environmental criteria in both groups, it can be confirmed that aesthetic elements positively affect other criteria in this experimental environment. Especially the participants in the driver group gave a strongly positive attitude.

#### 4.4.4 Limitations and future work

There are some limitations related to participants. The limitation is that participants feels discomfort while wearing the VR device (12.50% for the pedestrian group and 18.75% for the driver group, respectively). Since this research does not consider the dizziness during the experiment, more sample is required to get a less negative effect on the discomfort. Besides, due to the limitation of COVID-19, the sample size cannot be fully guaranteed, which may have a specific impact on the accuracy of the experimental results. Taking into account the adaptability of college students to the new VR technology, the selected participants are generally young. In addition, because of the random assignment of respondents groups, a wide gender split existed, which may impact the final results. Therefore, as an inclusive public space, the street should meet the needs of all users. More participants of different age groups and the effect of gender difference on the indicator preference should be considered in further study. With respect to quantitative analysis of safety performance, although the results show that people were optimistic that shared streets could improve safety, there was a lack of systematic safety quantitative analysis. It could be helpful for further research if more traffic simulation software can be added and conduct a safety analysis from a micro perspective.

For future work, additional scenarios related to traditional street mode can be added to longitudinally compare people’s subjective perceptions of this novelty shared conception. The difference between subjective collusion and objective results (e.g., time-to-collision, trajectory data) could be analyzed to provide more valuable design guidelines for city planners. In addition, more types of streets users should be considered in the experiment to develop the potential of shared streets. The street facilities that can provide convenience for the elderly, children and the disabled can be added to streets design and test using this analysis framework in further study.

## 5. Conclusion

This study aims at exploring the applicability and recognition of shared streets in China from both pedestrians’ and drivers’ perceptions. Taken an approximately 832m long street in front of Suzhou Museum as a case, Virtual Reality and HITL technology are utilized in this research. This study develops three street layouts that incorporate the Chinese environment, in which the road facilities and landscape layout adopt the Chinese style. The entire virtual environment can be highly visualized by making some improvements and adjustments to this system and adding more software collaborations. With the simulation experiment from both driver and pedestrian perception, responses regarding shared streets’ opinions and design preferences are collected for the following analysis.

The separation of sidewalks and carriageways is the fundamental difference between the three scenarios. Scenario A removes all delimitations, scenario C has a relatively high separation of people and vehicles, and scenario B has both A and C characteristics. Although the perception of this design varies significantly due to individual differences, high recognition is represented in all three scenarios in terms of amenity and safety criteria. The comprehensive evaluation shows that the participants in the pedestrian group gave similar scores for scenarios B and C, and are much higher than A. The driver preferred scenario C and gave the lowest score for scenario B in the other experimental group. This result presents that pedestrians have a high degree of recognition for the design of people-car sharing while drivers have reservations about the design that create more uncertainties for the driving process. Combining the two groups of participants’ perceptions, scenario C with the maximum soft isolation can get the relative highest support in this condition.

Furthermore, there is complex interrelationship existed among different criteria. The results reveal that aesthetic street furniture is also a decisive element to enhance safety and improve user satisfaction when using the street. The rational setting of the ground frontage and the spatial treatment of paving can also positively impact improving street safety and stimulating vitality. The suitable combination of vertical aesthetics and environmental utilization can sensuously and visually increase the uncertainty of the street environment, thereby enhancing street users’ vigilance.

## Supporting information

S1 File (ZIP)
