# Supplementary material for: Are shared streets acceptable to pedestrians and drivers? Evidence from Virtual Reality experiments
Source: PLoS One. 2022 Apr 15;17(4):e0266591. doi: 10.1371/journal.pone.0266591 (PMC9012376; doi:10.1371/journal.pone.0266591)
Supplement: S1 File — (ZIP) [file pone.0266591.s001.zip › supprting information/Manuscript-edited.docx]

# Explore the applicability of shared streets with virtual reality technology: A case study of Suzhou, China

**Lurong Xu (first author)**

Jiangsu Key Laboratory of Urban ITS，Southeast University;

Jiangsu Province Collaborative Innovation Center of Modern Urban Traffic Technologies, Southeast University;

Monash Institute of Transport Studies, Department of Civil Engineering,

Monash University, Clayton, Victoria 3800, Australia.

Email: 782145216@qq.com

**Taeho Oh (second author)**

School of Transportation

Southeast University, Nanjing, Jiangsu, 210096 China.

Monash Institute of Transport Studies, Department of Civil Engineering,

Monash University, Clayton, Victoria 3800, Australia.

Email: taeho.oh@monash.edu

**Inhi Kim (third author)**

Ph.D., Senior Lecturer

Department of Civil and Environmental Engineering Kongju National University, South Korea

Institute of Transport Studies, Department of Civil Engineering

Monash University, VIC 3168, Australia

Email: inhi.kim@kongju.ac.kr

**Xiaojian Hu (Corresponding Author)**

Ph.D., Associate Professor

Jiangsu Key Laboratory of Urban ITS，Southeast University；

Jiangsu Province Collaborative Innovation Center of Modern Urban Traffic Technologies, Southeast University；

School of Transportation, Southeast University

Southeast University Road #2, Nanjing, 211189, China

Email: huxiaojian@seu.edu.cn

**ABSTRACT**

While the development of cities tends to focus on improving traffic mobility, it has gradually neglected people’s demand for safety and comfort walking on the streets. To address this problem, shared streets that can integrate traditional street life and traffic mobility are getting more attention as pedestrian-friendly development. In order to measure the performance of shared streets, it is essential to identify how people feel when driving and walking around. However, investigating the various factors that influence the real world is not simple because of cost, time-consuming, and safety problems. Virtual reality and the Human-in-the-loop(HITL) have become useful tools for conducting experiments without compromising them. The experiments are performed on both pedestrians and drivers’ sides. The three shared street layouts in a virtual environment are designed according to the real shared street cases implemented in Europe. For the evaluation of shared street effects, questions in five aspects which are amenity, walking or driving experience, safety and environmental are asked to participants, respectively. MPR, EWM, and Fuzzy Comprehension Evaluation method are used to assess the performance. The result revealed that different groups of people have different sensitivity and preferences for each evaluation criteria but comprehension evaluation results showed that on the basis of shared street design elements, the scenario C with the largest soft isolation is more preferable in both pedestrian and driver’s group. The city planners can get helped through this shared street analysis where the new design and layout of the shared street could be tested in advance.

**Keywords:** Shared Streets, Virtual Reality, Human-in-the-loop, Unity 3D

## INTRODUCTION

Previously, commercial and tourist areas were regarded as living spaces where people could hang out and enjoy their time. However, with urbanization and the increased number of vehicles, these spaces have been separated people from the vehicles to keep street users safe and traffic efficiency. Even though the areas are separated by the traffic facilities to protect pedestrian from the vehicle, people still feel nervous about high speeding cars driving right next to the pedestrian area. Besides, people walking on the pedestrian street could get injured since speeding vehicles can penetrate traffic safety facilities and invade pedestrian zones.

In China, there are a lot of tourist and business districts that still did not take into account the accessibility and amenity of the traffic, simultaneously. Many specific pedestrian lanes can improve the safety of pedestrians by restricting the entry of motor vehicles, but they cause inconvenience and traffic congestion in the surrounding area. As an alternative way, in Netherland of the late 1970s, there has been an attempt to slow down vehicles naturally without depriving the road users of either motor vehicles or pedestrians of their right to travel. The concept of a shared street called Woonerf, which is the coexistence of vehicles and pedestrians on the same street layer, was introduced. Woonerf is one of the techniques for traffic calming that yield to each other by putting cars and pedestrians into the same street layer. This method consists of three factors that help coexist with cars and people. The three factors are street environment design, traffic facility deployment, and humans. The most essential element is the human being. It is because each person would be able to feel different levels of walking safety environment and driving environment depending on the design and location and type of transportation facilities. Therefore, it is necessary that the systemic shared space development analysis investigates the effect that considers human factors. Besides, the application of real street has various problems, such as long-term construction time, large budget requirement, and the risk of collision of pedestrians and vehicles when the improvement effect is insufficient.

In order to minimize these risk factors, this study utilizes virtual reality (VR) and Human-in-the-loop (HITL) technology. VR technology is a computer vision technology that enables people to experience the artificially created a virtual world in an immersive way. HITL technology allows human-computer interaction and fills the gap that the computer cannot implement with human factors, resulting in simulation results that consider human factors. A simulator platform that integrates the two technologies is be established from a pedestrian’s and driver’s point of view. By using this simulator platform, the experiment about the different designs and strategies on shared space is checked through a practical case study.

This research start with the curiosity: What is the acceptance of shared streets in the street environment based on Chinese culture and which shared street condition is preferred? Does aesthetic design elements have correlations to improve street safety? Do drivers and pedestrians have different feelings of use in the same environment？

This paper is divided into five parts. The second section reviews the concept and safety consideration of shared streets. Besides, the application of VR and HITL technology is involved in this part. In the third section, the layout and design philosophy of scenario settings, the building of the virtual environment, the procedure of simulation experiment, questionnaire survey, and quantitative evaluation are described in detail. Section 4 presents the explanation and discussion of the result from the simulation experiment. The last section concludes the experiment results and proposes suggestions for the future development direction.

## LITERATURE REVIEW

### Shared Streets

Karndacharuk, Wilson [1] explained the shared space with the fundamental differences between traditional mixed streets and shared streets. In shared streets, traditional street facilities such as curbs, signs, and signals are replaced by integrated and people-oriented public spaces. This measure will encourage social interaction, walking, biking, accessibility, and reduce the speed of motor vehicles. In comercial districts and residential areas, shared streets can play a huge potential. Through public seating, artwork and landscaping, it can greatly stimulate the vitality of urban life, as an extension of the front yard, and create a livable community life [2]. In order to make the role of shared space efficiently, deploying design elements on the shared streets is necessary [3]. In aesthetics, acoustics and environmental science perspective, color design elements can play an important role in calming the driving behavior on the street. Besides, coloring on the street components helps to distinguish the different roles of streets, such as the safe pedestrian zone and to give warning of caution factors, such as tunnels and barriers[4]. Hamilton-Baillie [5] presented safer and more vibrant streets by reshaping the experience in the urban area through public arts. Biddulph [6] insisted that the rational layout of aesthetically creative transportation facilities would be able to maximize the advantages of shared streets under the premise of considering the social-cultural background.

When it comes to the safety of the shared street layout, some people remain skeptical attitude about the new layout since it removes all the physical factors between pedestrian zones and streets compared to conventional street layout [7]. However, the ultimate objective of the shared street is to make the “mental speed bump” effect through the interaction of the human-vehicle by blurring the boundary between street for the cars and pedestrians. The mental speed bump effect intends to ask the driver to put more attention in situations that pedestrians freely cross the shared street [8]. Ruiz-Apilánez, Karimi [9] showed that shared streets are capable of preventing traffic accidents through comparison between traditional streets. Obeid, Abkarian [10] investigated the yielding behavior between drivers and pedestrians in the mixed street from the driver’s perspective using a driving simulator. The result showed that the yielding rate was higher in the shared street. The additional result based on the Kruskall-Wallis method showed that the behavior of participants driving close to pedestrians was statistically less aggressive, particularly on the streets with low-speed limits.

Regarding the qualitative assessment of shared street, Ruiz-Apilánez, Karimi [9] described the layout and performance of different shared streets in six areas from the survey. It summarized how these different spatial layouts affect street safety, amenity, and the distribution of activities to the street users. Charlton, Mackie [11] recognized that the perceptual limits of pedestrian areas vary depending on the street layout, which shows that street design is a powerful tool to change the built environment. Using the result from the surveys and expert interviews, Karndacharuk [12] presented five different evaluation standards on shared streets, such as place, pedestrian, vehicle, economic, safety. The five criteria were used for the significant pedestrian interaction quality improvement, vehicle speed reduction, confidence in space dominance, and revitalize land use for successfully shared space operations.

However, there is few previous studies focus on systemic analysis that can comprehensively consider various human factors. The existed literature usually conducted evaluation from a single perspective (drivers or pedestrians) and the existing literature on shared streets rarely discusses the streets performance from two aspects simultaneously, and combines the two perceptions to get the final evaluation.

### Virtual Reality

Since virtual reality technology becomes accessible, it has been widely used in various fields [13]. Lok, Ferdig [14] applied virtual reality technology to medical education. Tsai, Hsieh [15] used VR technology to orthopedic surgery simulator that illustrates the successful implementation in virtual surgery. Meggs, Greer [16] found that interior design teaching with VR technology could significantly enhance student participation and learning output. Portman, Natapov [17] recognized the use of VR in architecture and landscape.

However, Virtual Reality technology is still in a stage of continuous development, and there are undoubtedly some shortcomings in popularization. For example, Anthes, García-Hernández [18] raised the issue of the limitation of hardware devices. During the experience, the quality of the equipment is directly related to the effect of the immersive experience, a lot of financial support is needed. Besides, due to the lag in the interaction between screen imaging and the user, the gap between the 3D image and the real image through the eyepiece, and the discomfort of wearing a helmet, it will bring negative effects to users such as dizziness and headaches [19]. At the application level of education and training, VR technology is not suitable for all conditions, especially for older teachers whose teaching methods are difficult to change. If they cannot adapt well to the difference between VR technology and traditional teaching methods, this kind of discomfort will make it difficult for this new VR teaching mode to play a positive role [20].

Although the immature technology development limits the application of VR technology, it has huge potential and role in traffic simulation. Meir, Oron-Gilad [21] simulated the scenario of crossing road, and let the child participates engage in the virtual environment experiment. Through the risk assessment test, he tested the risk perception ability of children in this traffic environment from the perspective of pedestrians. Chun, Ge [22] proposed a method that can use VR technology to convey the design plan to city planners, verifying the significant role that VR technology can play in transportation planning. In addition, the immersion of VR technology is unmatched by pictures and text information. In response to the ethical dilemma of autonomous driving, Sütfeld, Ehinger [23] used VR technology and text-based surveys to test the decisions made by different participants in the face of different factors. The entire experiment further verifies the application of VR technology in the field of autonomous driving.

The above researches confirmed the immersion and interactivity of the VR technology and demonstrated its potential in realizing realistic surrounding environments for experiment.

### Human-in-the-loop

Human-in-the-loop (HITL) with VR is defined as a model that plays a vital role in the simulation due to human-computer interaction in real-time impacts on research results [24]. The HITL has been widely used in the evaluation of the design industry and the exploration of environmental science. Kefalidou, D'Cruz [25] focused on the degree of comfort. User-centered design of airport interior through 3D visualization and emotional test for passengers verified the value of HITL with VR in practice and operation. Jiang, Masullo [26] conducted an experiment using VR through simulating street-design scenarios to explore the influence of soundscape from a human perspective.

In the field of traffic engineering, there is plenty of research that proves the applicability of HIIL. In order to investigate the traffic safety in pedestrian's perspective, Deb, Carruth [27] developed a pedestrian simulator to investigate the safety issue in signal intersections. Lei, Yalian [28] established a driving simulator with the Unity 3D game engine to test the fuel consumption of hybrid electric vehicles. Hartmann, Viehweger [29] examined the anti-collision system for autonomous vehicles to protect the pedestrian's safety. Oh [30] proposed an emerging platform named Human in Virtual Reality in the Loop Simulator (Hi-VRiLS) and verified that human factors need to be considered in safety analysis simulation.

In the field of transportation, HITL technology is widely used in traffic safety research, but there are rare papers considering human factors by this technology. Moreover, the shared streets research coporating with VR technology is still missing. Therefore, shared street research based on HITL model and VR technology is meaningful to look into the human interaction in simulation.

## METHODOLOGY

This research is divided into two sections, as shown in Figure 1. The first section is to build a virtual experiment environment of shared streets according to the different design and deployment of traffic facilities. Three scenarios are selected for the analysis based on the experience of shared space projects in various countries. The second section is to assess the user reactions depending on roles in the differently built shared street environment.

In the first step, the components such as background environments, pedestrians and vehicles were built. Sketchup and Blender are utilized to establish the experiment background environments. Building and streets built in Sketchup are imported into Unity3D, and Blender is used for adjustment between Sketchup and Unity3D if there is a rendering error. After that, Substance Designer and Painter tools are used to handling the texture and materials on the building walls and street components for better realistic scenery. In order to create the pedestrian models, Adobe Fuse is used for creating the basic human appearance and outfits to Mixamo for allocating the movement to each pedestrian model. Lastly, all the buildings and pedestrian models are imported in Unity 3D to have an actual street environment. For the driving experience, VR devices and driving equipment, such as head-mounted devices, steering wheel, and pedals are included. A complete traffic system is also implemented using scripting, in which pedestrians and vehicles can interact and yield each other in a virtual scene. This script mimics the reality of simulation to the greatest extent and can improve the accuracy of the experimental results.

In the second step, an experiment and questionnaire survey about the experience of shared streets are conducted for a performance assessment from both pedestrians and the driver’s point of view. The total of thirty-two volunteers joined in this experiment and are divided into two groups. After finishing the experiments, all the participants are asked to the shared streets experience about three scenarios and VR device experience.

Based on the collected data from the questionnaire, the Median Perception Rating (MPR) measure is adopted to evaluate the performance of three scenarios following different criteria, Entropy Weight Method (EWM) and Fuzzy Comprehension Evaluation method are used to integrate all the criteria for making sure a comprehensive assessment.


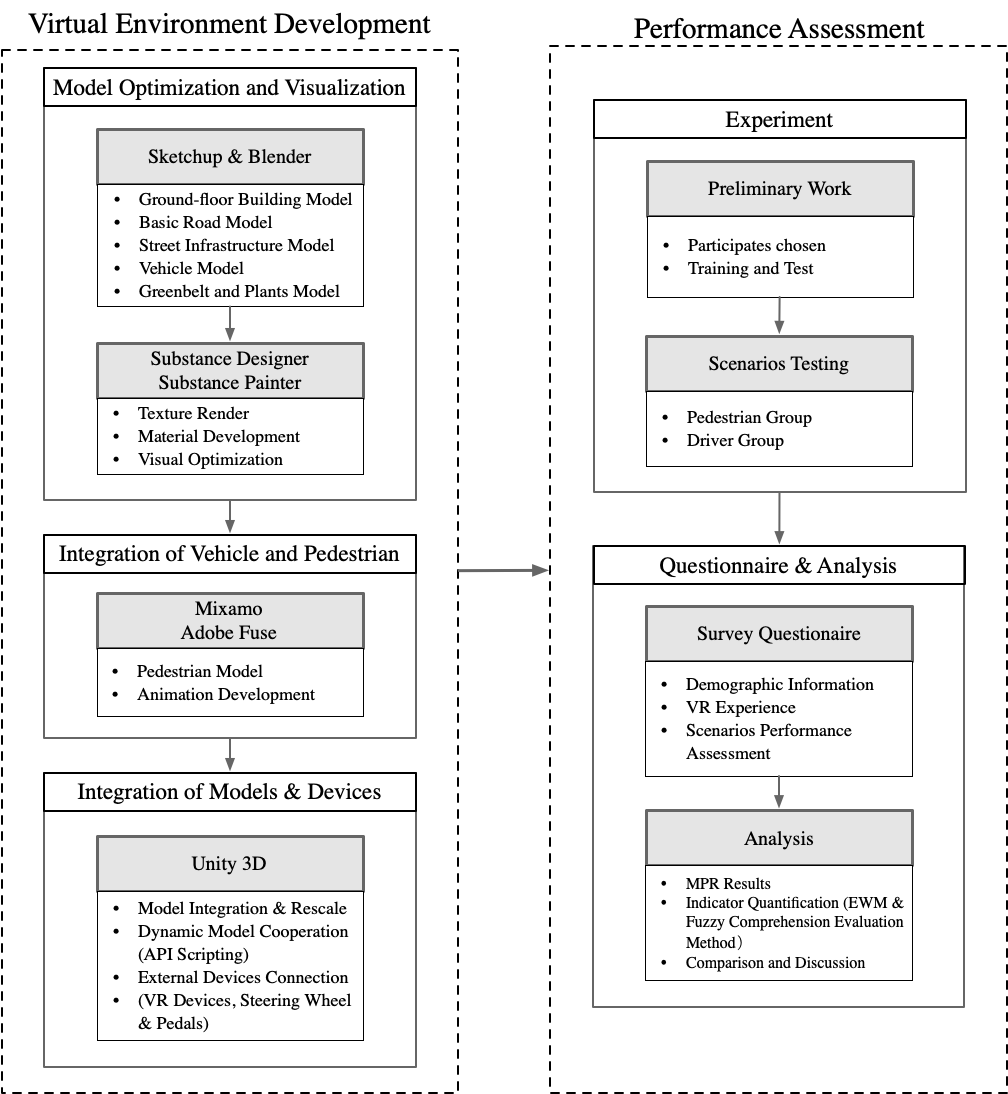


Figure 1 Framework of experiment development

### Scenario Setting

For the shared street experiment, the 800 meters long straight street where the Suzhou Museum (see Figure 2) is was selected. Suzhou Museum is the most attractive tourist destination in Suzhou, China and attracts plenty of visitors every year. However the road condition around the Suzhou Museum is lagged. Therefore, the street is often suffering to take the huge tourism demand causing the traffic congestion. Therefore, dangerous conflict occasion between pedestrian and vehicles happens frequently. In order to address traffic congestion, this street was transformed into a specific pedestrian section since 2018. This transformation has caused inconvenience to the residents that vehicles have to make a big detour to avoid this area. In addition, the Humble Administrator’s Gardens is located right next to the Suzhou Museum, and various shops are located along the other side of the street. Therefore, it’s a suitable segment as a case study to investigate the effectiveness of shared street regarding it is a good way to combine mobility and walkability as well as stimulate livability and commercial development. Based on the existed street layout, three different street layouts are established.


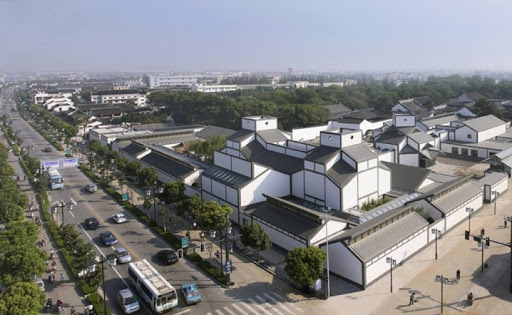


Figure 2 Suzhou Musume

#### Scenario A

According to the concept of Woonerf, scenario A is a simple shared space with homogeneous pavement and without any design factor on the street so that pedestrians and vehicles can freely move around (see Figure 3.A). This scenario aims to strengthen the “mental speed bump” effect that encourages human-vehicle interaction by removing facilities that split vehicles and pedestrians on the street. This concept intends that pedestrians freely crossing the street bring uncertain environmental prompts to the driver. By giving pedestrians more freedom on the streets, the majority of users in street spaces can be transferred from vehicles to pedestrians. Pedestrians are expected to get more guaranteed priority to travel the whole area [31].

Besides, the unified and continuous street pavement can change the overall atmosphere of the space and provide people with places to stay and play. The uniform paving can also improve comfort for pedestrians to increase attractiveness so that pedestrians gradually become the protagonists of the street user while vehicles become as intruders. By doing this, drivers put more attention on the streets while driving to avoid accidents with people in shared space that can happen various activities at any time.

#### Scenario B

Following the space reconstruction case of Garibaldi-Brera Environment Island [32], scenario B includes traffic calming factors combining with the traditional Woonerf concept (see Figure 3.B). In addition, the aim of traffic safety measures has shifted from merely restricting speed and flow to actively creating harmony between people and vehicles by making shared street dynamics according to Mackie, Charlton [33]. Based on this knowledge, scenario B intents to ask drivers to drive slowly unintentionally for harmony between drivers and pedestrians since the people are more likely to behave more cautiously when they are exposed to complex, attractive, and uncertain environments.

When it comes to the pavement, the red paving material that is much softer than the gray-colored road material is applied. By doing this, vehicles on the red road slow down naturally since they get the frictional force. In addition, the driving path guides the cars to drive the S-shape of the street that reduces the driving speed dramatically.

Facilities and environmental elements on the streets enrich the scene and enhance the psychological satisfaction of users. The trees are located in the opposite direction of the vehicle traffic path to create the effect that a pedestrian zone delimited by trees and facilities that make people enjoy their time on the streets, such as tables, chairs, and stores.

#### Scenario C

The pedestrian zone is placed parallelly to the straight roadway and is separated by plants and facilities based on a design of the South Kensington [9] (see Figure 3.C). In addition, there are spaces for parallel parking along the street. A number of public facilities for the pedestrian are located to make people stay longer on the street. The advantage of this is to increase the sense of walking environment safety by dominating the area, increasing the number of walking activities [34].

The most considerable difference from the previous two scenarios is that scenario C sperate the role of the street using street facilities and different pavement without changing the linear driving way.


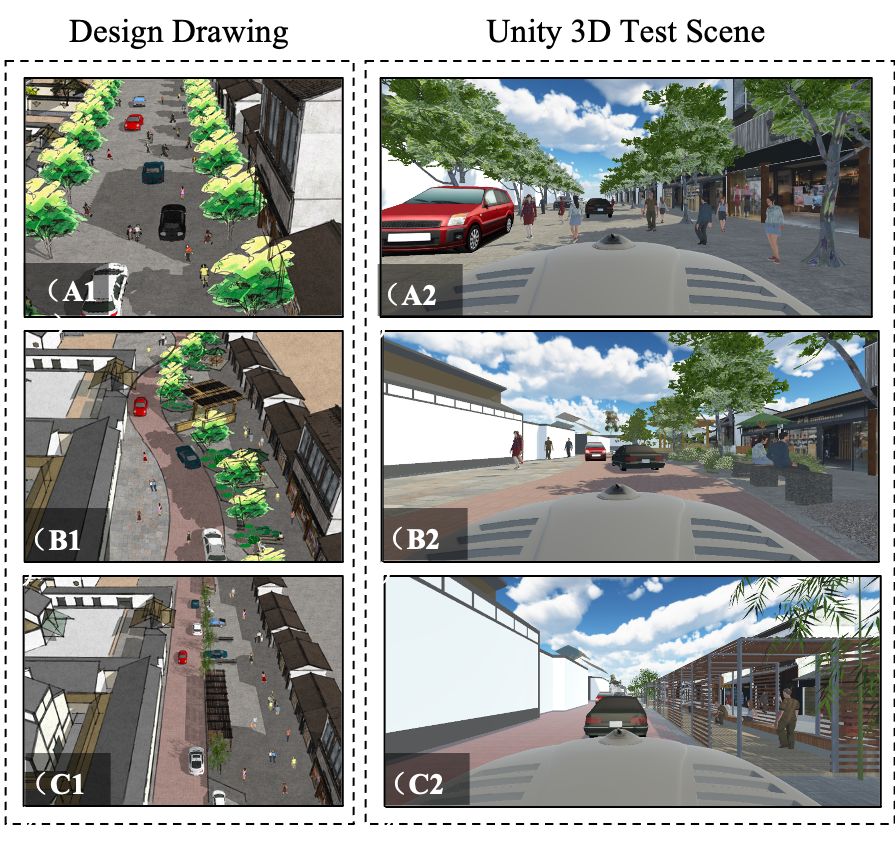


Figure 3 Scenarios Setting

### Virtual Environment Development

#### Experiment Environment Establishment

Several applications are used to make a virtual experiment environment consisting of static and dynamic models. When it comes to static models, the structure of base models was created using SketchUp that allows to make buildings and street segments. Once the sketch of the shared street layout was done, Blender, which is an open-source modeling software, was used for model optimization since it has powerful rendering functions. Besides, given the ability to interact with other visual modification software, Substance Design and Substance Painter was able to cooperate with Blender as well as Unity 3D successfully. Furthermore, the entire workflow referenced from a game development remarkably achieved visual fidelity and conformed to the characteristics of the aesthetic development.

Figure 4 shows the detailed visualization created by the workflow. Considering the texture of the paving material had an essential influence on the realization of the concept of street layout in this research, Substance Painter was used to achieve visual authenticity and aesthetics. Simultaneously, Substance Designer focused on the material restoration when driving on the street, the feel of the material could convey feedback to the participants through the steering wheel (see Figure 4-A). Moreover, high-definition textures were used to achieve a high degree of overlap with the real environment (see Figure 4-B Suzhou Museum, Figure 4-C Commercial Stores, and Figure 4-D Street Furnitures, respectively).


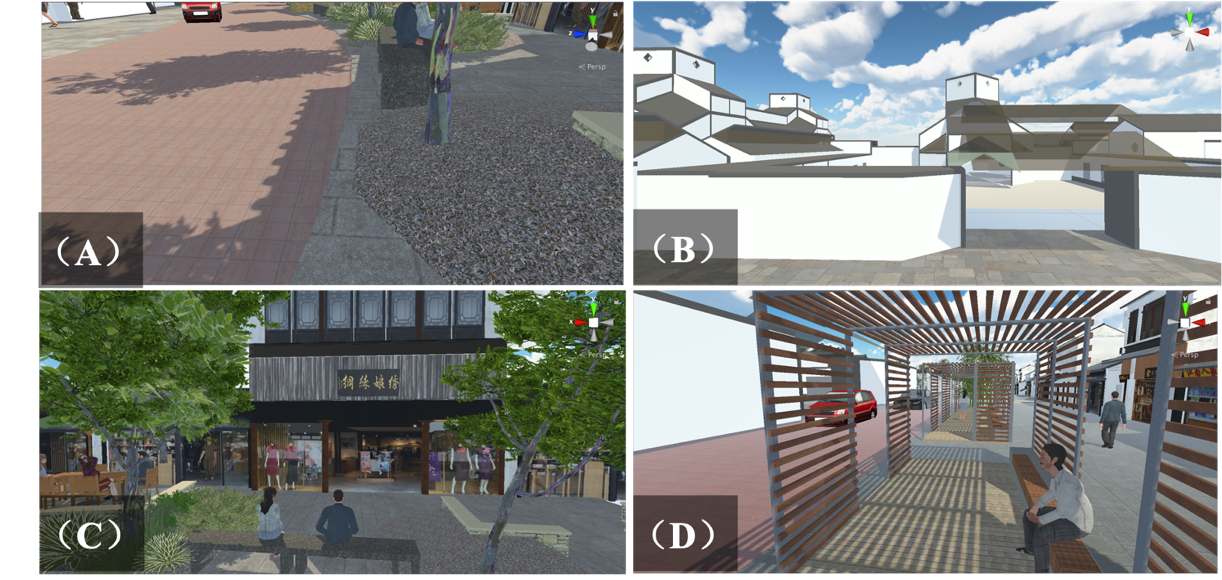


Figure 4 Visualization in Unity 3D

#### Street Users setting

In order to make a realistic street environment, the people and cars were controlled by computer programming from the asset package in Unity 3D called Urban traffic system 2018. They randomly walked and drove around naturally. Considering the similarity between the geographical location of this case and the Exhibition Street-Museum mentioned in [9], the traffic volume and speed of each scenario were 600veh/h and 30 km/h, which is the actual speed limit in the area. Besides, the volume and walking speed for the pedestrians were 300ped/h and 5 km/h, respectively. It is noteworthy that when the distance between two street users is less than two meters, they yield each other.

Moreover, Mixamo was used to give animation to the fixed pedestrian model. The people in the virtual world behaved more realistic by walking, laughing, talking, and sitting.

### Experiment

This experiment was carried out in Suzhou, China in January 2020. All the participates are postgraduate students at Southeast university-Monash university Joint Graduate school. This experiment was approved and authorized by Southeast University. We also introduced the voluntary statement on the first page of the survey questionnaire^[[1]](#footnote-1)^. The survery is completely CONFIDENTIAL, ANONYMOUS, and VOLUNTARY. The detailed process of integrating Unity 3D and VR equipment is described in [30]. After choosing participates, this experiment can be divided into five steps: introduction, grouping, testing, scenario experiment, and questionnaire survey.

In the first step, the concept of shared streets was briefly introduced to the participants. Then, all thirty-two participants were divided into two groups randomly. Each group had sixteen people. The participants in group one experimented with a driver using driving devices, including steering wheel and pedals. The participants in group two played a pedestrian role using gaming controllers. In the third step, all the participants were required to adopt the VR environment and experimental equipment in a test scenario until being familiar with the VR environment and controlling before the actual experiment. In step four, each participant was required to experiment with three scenarios. Participants got three minutes of break between the scenarios. During the experiment, each participate in the driver group took an average of fifteen minutes while participate in the pedestrian group took an average of thirty minutes. In the end, all participants were required to answer the questionnaire.

Figure 5 shows the simulation environment. To be specific, A-1 and A-2 show the environment in reality and scene in Unity 3D of the driver group, and B-1 and B-2 show the environment in reality and scene in Unity 3D of the pedestrian group.


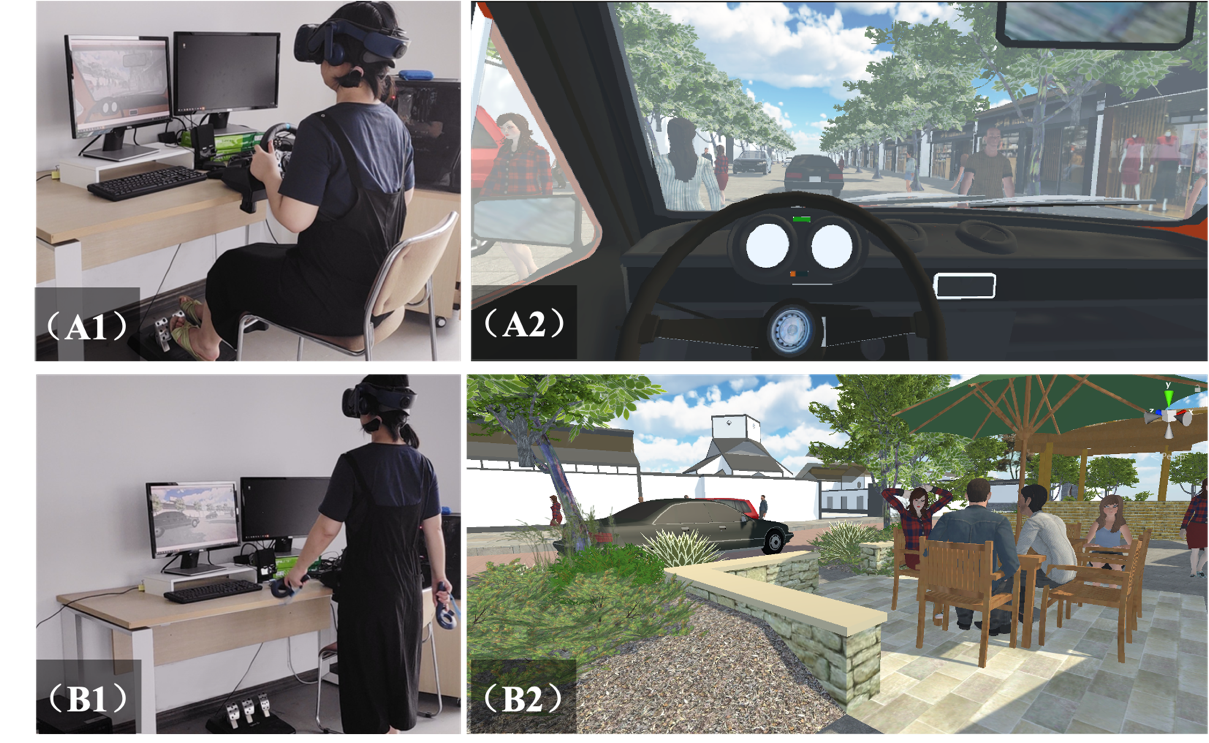


Figure 5 Experiment Environment

### Questionnaire Survey

In order to conduct a performance assessment, pedestrian satisfaction factors were considered, such as ‘Pedestrian Environment Review System (PERS)’ and ‘Pedestrian Environment Data Scan’ [35, 36]. Based on the 6-point Likert rating scale proposed by Bradburn, Sudman [37], the evaluation criteria range is from ‘-3’ to ‘+3’, representing ‘strongly disagree’ and ‘strongly agree’. This scoring method deliberately removes the neutral “0” point, so that participants can be more cautious when scoring, making the data results more reliable and more intuitively reflecting positive and negative evaluations. In addition, the level of satisfaction of shared street was evaluated in five different aspects following Karndacharuk [12].

The questions divided into two types: group and common questionnaire (see Table 1). Participants in the different groups were asked to answer to their shared space experiences according to their roles. The group questionnaires included five criteria, such as comfort, freedom, safety, habits, and environment. After that, the common questionnaire to know awareness of shared streets and VR experience were asked.

Table 1 Design of Questionnaire Survey

| **Group Questionnaire (scale from -3 to +3)** | | |
| --- | --- | --- |
| **Criteria** | **Pedestrian Group** | **Driver Group** |
| Comfort | I felt comfortable walking | I felt comfortable driving |
| Walking/Driving Experience | I could freely move around | I could drive smoothly |
| Safety | I felt safe and secure in the street. | I would like to drive slowly |
| Economic/Priority | I enjoyed time in the street | I preferred to yield to pedestrians. |
| Environment | I felt the traffic noise is reduced compared to conventional streets. | |
| **Common Questionnaire** | | |
| Have you ever heard of the shared street before? (a)Yes, (b)No. | | |
| Have you ever experienced VR before? (a)Yes, (b)No. | | |
| Do you ever feel dizzy using the VR driving simulator? (a)Yes, (b)No. | | |
| Your opinion of shared street’s layout compared to conventional streets. | | |

### Quantitative evaluation method

For the evaluation, Median Perception Rating (MPR) proposed by Torgerson [38] is used as a qualitative indicator to analyze the performance. The same qualitative assessment method is leveraged by Karndacharuk, Vasisht [39] in the before and after transformation case analysis of shared streets.

The detailed process of MPR method can be concluded by the following steps:

Step 1: Organize the data collected from the questionnaire survey and calculate the proportion of respondents in each score rate.

Step 2: Conduct cumulative calculation in accordance with the response proportion; each score rate is used as the upper boundary.

Step 3: Plot the cumulative curve in terms of the calculation results in step 2 in which the abscissa is the score rating from “-3” to “+3” and the ordinate is the proportion value from 0 to 1.

Step 4: By determining the point of the curve at 0.5 on the ordinate, find the value corresponding to the abscissa, the MPR value can be obtained.

Entropy Weight Method (EWM) method is an objective weighting method, which can be used to evaluate the influence of a certain index on a comprehensive evaluation. The following formula presents the procedure to conduct the EWM method:

Step 1: Define the number of samples as n, number of criteria as m, use Equation 1 to conduct normalization. Where, $x_{ij}$ represents the value of sample $i$ for criteria $j$ ($i=1, \ldots, n;j=1, \ldots, m)$.

$v_{ij}= \left\{ \begin{aligned} \frac{x_{ij}-\min\left( x_{j} \right)}{max\left( x_{j} \right)-min\left( x_{j} \right)} \\ \frac{max\left( x_{j} \right)-x_{ij}}{max\left( x_{j} \right)-min\left( x_{j} \right)} \end{aligned} \right.$ (1)

Step 2: determine the eigenvalue proportion of sample $i$ under the evaluation criteria $j$ using Equation 2:

$p_{ij}=\frac{v_{ij}}{\sum_{i=1}^{n} v_{ij}}$ (2)

Step 3: the calculation of information entropy of sample $i$ under the evaluation criteria $j$ using Equation 3:

$e_{ij}=-\frac{1}{ln\left( n \right)}\sum_{i=1}^{n} p_{ij}ln\left( p_{ij} \right)$ (3)

Step 4: Define the diversity factor using **Equation 4**:

$d_{j}=1-e_{j}$ (4)

Step 5: Calculate the weight of each criteria using **Equation 5**:

$w_{j}=\frac{d_{j}}{\sum_{k=1}^{m} d_{j}}$ (5)

Fuzzy Comprehension Evaluation method can transform qualitative evaluation into quantitative evaluation based on the membership theory.

Step 1: Define the number of samples as n, number of criteria as m. Establish a comprehensive evaluation criterion set $U=\left( u_{1},u_{2},\ldots, u_{m} \right)$, and a comprehensive evaluation grade set $V=\left( v_{1},v_{2},\ldots, v_{n} \right)$.

Step 2: Build a fuzzy relationship matrix using **Equation 6**:

$R=\left[ \begin{aligned} R| u_{1} \\ R| u_{2} \\ \cdots\\ R| u_{m} \end{aligned} \right]=\left[ \begin{aligned} r_{11} r_{12} \cdots r_{1n} \\ r_{21} r_{22} \cdots r_{2n} \\ \cdots\cdots\cdots\cdots\\ r_{m1} r_{m2} \cdots r_{mn} \end{aligned} \right]$ (6)

Step 3: Determine the weight vector of criteria by AHP or EWM method and represent it as $A=\left( a_{1},a_{2}, \cdots,a_{m} \right)$.

Step 4: Build a comprehensive evaluation model using **Equation 7**:

$A\circ R=\left( a_{1},a_{2}, \cdots,a_{m} \right)\left[ \begin{aligned} r_{11} r_{12} \cdots r_{1n} \\ r_{21} r_{22} \cdots r_{2n} \\ \cdots\cdots\cdots\cdots\\ r_{m1} r_{m2} \cdots r_{mn} \end{aligned} \right]=\left( b_{1},b_{2}, \cdots, b_{n} \right)$ (7)

## PERFORMANCE ANALYSIS

### Demographic characteristics

They are postgraduate students at Southeast university-Monash university Joint Graduate school in Suzhou, China. The first group of participants joined pedestrian scenario (Mean age 22.16 years, SD = 1.34, 43.75% males, 56.25% females) and the second group of them joined the scenario being drivers (mean age 22.97 years, SD = 1.16, 68.75% males, 31.25% females).

Table 2 shows the basic demographic information of participates, in which people who have not experienced the VR environment before this study accounted for the majority. However, only a small proportion of people reacted with dizziness during the experiment (12.50% for the pedestrian group and 18.75% for the driver group, respectively).

Table 2 Demographic Information

| **Items** | | **Experimental Group (%)** | |
| --- | --- | --- | --- |
|  |  | **Pedestrian** | **Driver** |
| ***Gender*** | | | |
|  | Male | 7 (43.75) | 11 (68.75) |
|  | Female | 9 (56.25) | 5 (31.25) |
| ***Age (Mean Value)*** | | *22.16* | *22.97* |
| ***VR Experience*** | | | |
|  | **Have not Experienced Before** | **10 (62.50)** | **9 (56.25)** |
|  | Have Experienced Before | 6 (37.50) | 7 (43.75) |
|  | Dizzy | 2 (12.50) | 3 (18.75) |
|  | Not Dizzy | 14 (87.50) | 13 (81.25) |

### Statistical Analysis

Two ANOVAs (Analysis of Variance), which is a significant test method for the difference between the means of two or more samples were conduct to test the significance of street design impact on five shared street ratings. The results show that the street design effect of three scenarios on ranking is significant at 0.05 confidence level in both experienment group (df=2, F=5.9175, p=0.0031 for pedestrian group and df=2, F=8.5825, p=0.0002 for driving group, respectively).

Spearman’s rank correlation matrix of two groups are presented in Figure 6, respectively. With respect to pedestrian experiment group, comfort criteria is highly related to walking experience in three scenarios. Besides, “safety” criteria is also affected by other criteria, and this interrelationship varies in different scenario. It is worth noting that the correlation coefficient between “safety” and “walking experience” is the highest value in pedestrian experiment group (0.744). While the correlation relationship among five criterias is different in driving group. The highest coefficient value appears between “comfort” and “priority” in scenario C (0.649). Moreover, complex interrelationship appears in scenario B that there are four pairs of criterias are statistically correlated. Comparing these two experiment groups, the coorelation among various criterias are both obvious in scenario B. Except for scenario C in driving experiment group, at least one criteria in each scenario is related to “safety”.


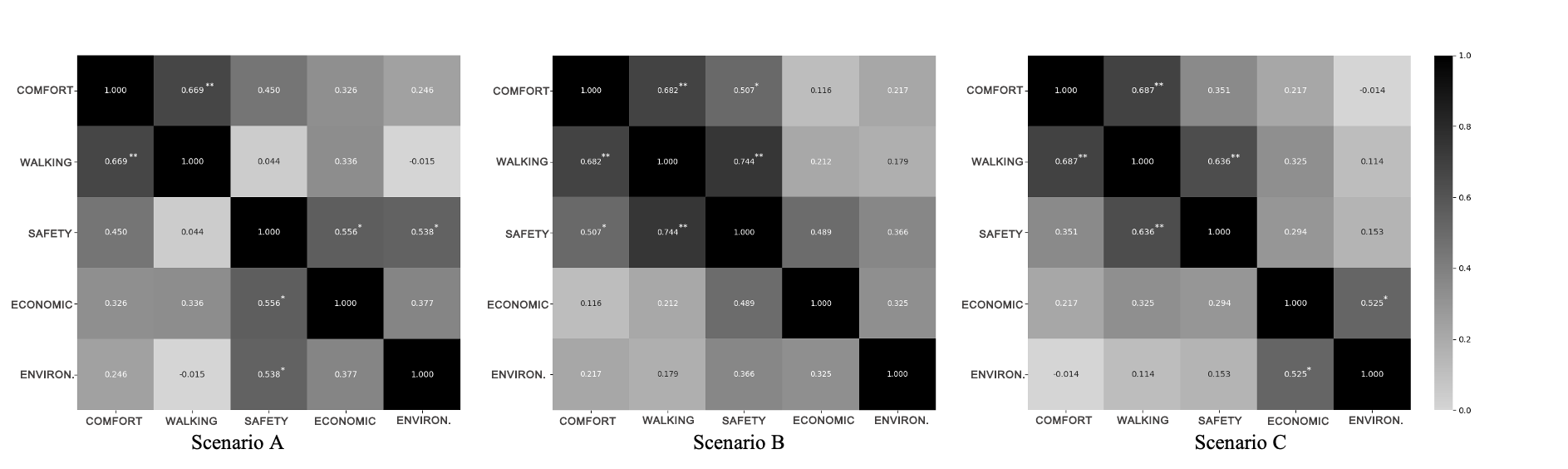


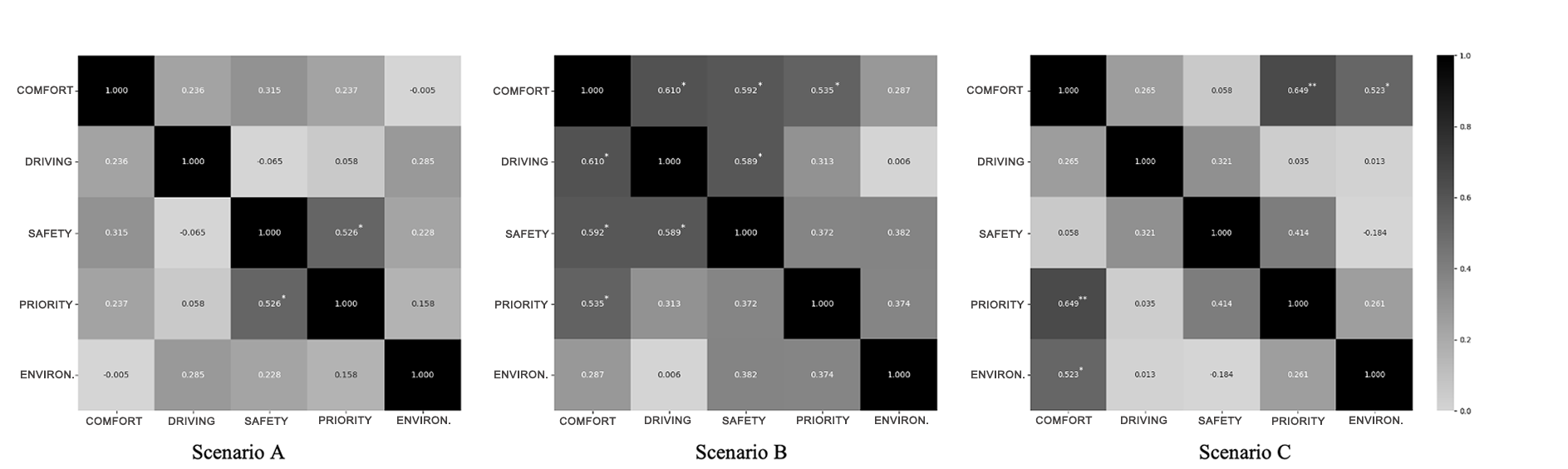


Figure 6 Spearman’s rank correlation matrix of pedestrian(a) and driver(b) experiment groups

### Quantitative evaluation

Table 3 is a summary of MPR, mean value, and standard deviation from a pedestrian and driver perspective. Plus, the Entropy Weight Method (EWM) and Fuzzy Comprehension Evaluation method are conducted to integrate the five criteria to a comprehensive indicator to quantitatively evaluate the overall performance of three scenarios (see Figure 7).

#### Pedestrian Group

Scenario B was better than the other two scenarios in the performance of the comfort and pedestrian criteria. In comparison, scenario C was the best in the performance of the following three criteria. It is noteworthy that the MPR value of scenario A remained the lowest among the whole five criteria. With regard to mean value, even though the performance of safety perception of scenario A represented higher value than scenario B, the other criteria show that the evaluation of scenario A was the lowest on the mean value. From the comprehensive evaluation result, scenario C had the highest evaluation among the three settings from pedestrian perception.

#### Driver Group

It is almost identical to the MPR value of scenario A distributed among the five criteria. Different from the result of the pedestrian experiment, the MPR results reveal that scenario C outperformed over the other two scenarios in comfort, driver, and environmental aspects. Besides, scenario B had a higher score in the assessment of safety and priority criteria. From the perspective of the comprehensive evaluation indicator, scenario C was almost identical to scenario B. Regarding the comparison of the pedestrian group, only the performance evaluation of scenario B form drivers’ perception outperformed over the other two settings.

Table 3 Median Perception Rating (MPR)

| **Index** | **Scenarios** | **Pedestrians** | | | **Drivers** | | |
| --- | --- | --- | --- | --- | --- | --- | --- |
|  |  | **MPR** | **Mean** | **SD** | **MPR** | **Mean** | **SD** |
| Comfort | A | 1.297 | 1.188 | 1.509 | 1.167 | 0.938 | 1.853 |
|  | B | **1.444** | 1.688 | 1.364 | 1.286 | 1.563 | 1.171 |
|  | C | 1.429 | 1.625 | 1.158 | 1.455 | 1.938 | 0.556 |
| Walking/  Driving Experience | A | 1.375 | 1.438 | 1.456 | 0.833 | 0.875 | 1.495 |
|  | B | **1.569** | 1.813 | 1.136 | 1.286 | 1.125 | 1.798 |
|  | C | 1.556 | 1.938 | 1.938 | 1.778 | 2.313 | 0.583 |
| Safety | A | 1.063 | 1.000 | 1.248 | 1.222 | 1.250 | 1.346 |
|  | B | 1.333 | 0.916 | 0.916 | 1.714 | 1.938 | 1.248 |
|  | C | **1.571** | 1.938 | 1.029 | 1.375 | 1.750 | 0.968 |
| Economic/Priority | A | 0.667 | 0.813 | 1.285 | 1.143 | 0.875 | 1.798 |
|  | B | 1.142 | 1.438 | 1.116 | 1.500 | 1.438 | 1.694 |
|  | C | **1.250** | 1.063 | 1.749 | 1.429 | 1.563 | 1.368 |
| Environment | A | 1.143 | 0.750 | 1.953 | 1.125 | 1.062 | 1.435 |
|  | B | 1.556 | 1.813 | 1.184 | 1.489 | 1.836 | 1.014 |
|  | C | **1.625** | 1.875 | 1.218 | 1.556 | 1.938 | 0.966 |


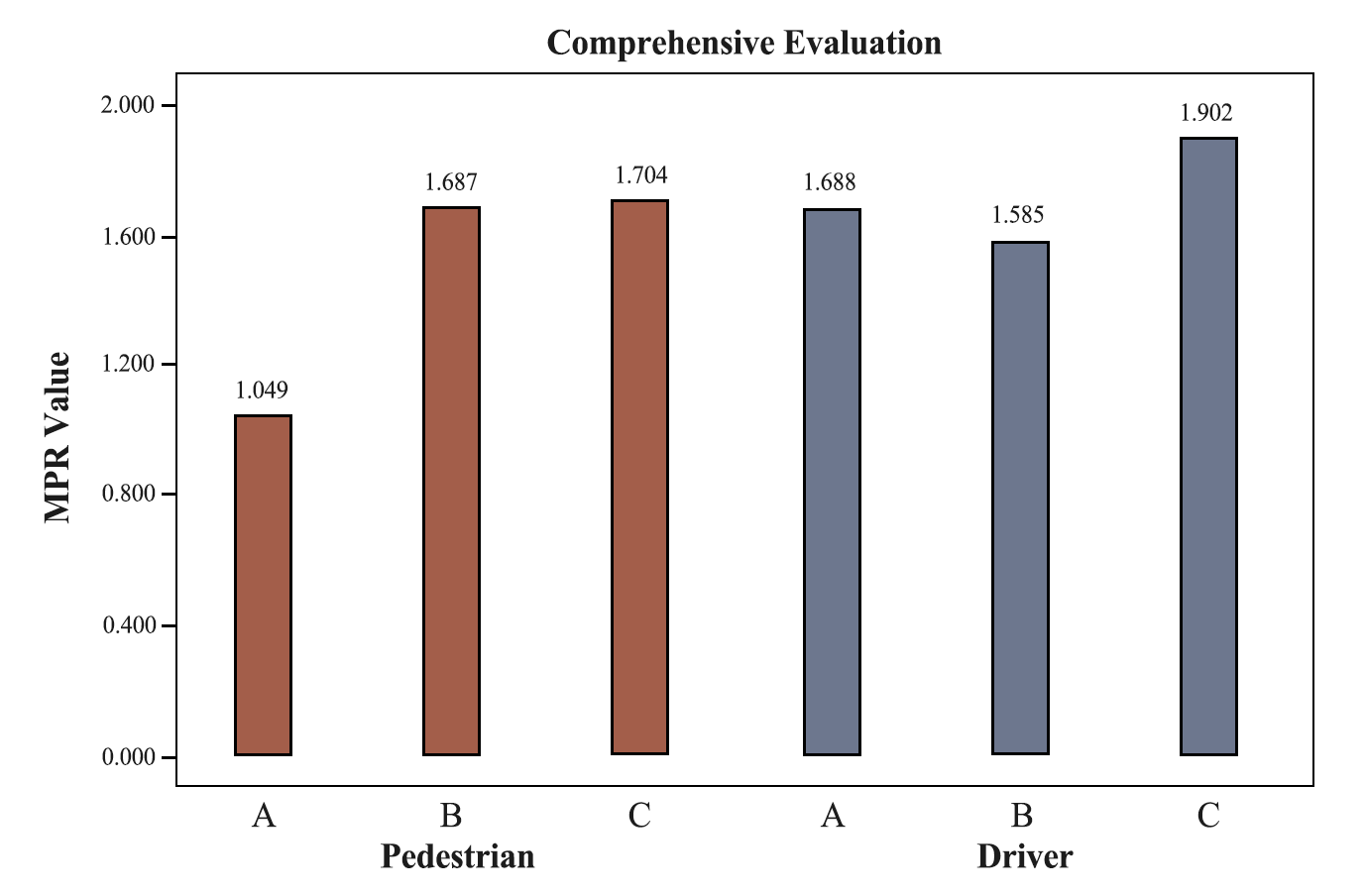


Figure 7 Comprehensive evaluation result

### Discussion

#### Recognition of shared streets

Although the aim of the street design is the harmonious coexistence of vehicles and pedestrians on the street, it might be possible that the degree of shared street awareness would vary due to the perceived differences of each other. To be specific, there was little difference in the MPR index between scenario B and C in the amenity level from the pedestrian group experiment while there were huge differences between scenarios A and B as well as scenarios A and C (0.147 and 0.132, respectively). In the driver group, the MPR and Mean value of scenario A in driving experiment criteria was significantly lower than the other two settings (MPR 1.167, Mean 0.938 for scenario A, MPR 1.286, Mean 1.563, and MPR1.455, Mean 1.938 for scenario B and C, respectively). Moreover, in common questions, the opinions in scenario A showed different responses compared to other scenarios. The comments like ‘chaotic’ and ‘disordered’ on scenarios A appears on the questionnaire survey of the driver group. It can be inferred that from drivers’ perception, completely removing all isolation measures will reduce their sensory satisfaction. However, according to the responses, the majority were optimistic about the shared street layout of all the three scenarios. The popular comments were that “I prefer this kind of street compared to conventional streetways”. Therefore, the optimistic opinion of the shared street is revealed.

#### The difference between pedestrian and driver groups

It is noteworthy that there was an enormous difference between the two groups regarding the MPR and means value. The MPR value presented in Table 3 reveals that the drivers’ ratings for these three scenarios were generally lower than pedestrians for the criteria of comfort, driving experience, and environment. For example, for the evaluation of walking and driving experience criteria in scenario A, there was a massive difference in MPR value between driving and pedestrian experiment (MPR 1.375 for the pedestrian group and MPR 0.833 for driver group). Besides, according to the result of the comprehensive evaluation presented in Figure 7, drivers prefer scenario A more than scenario B, while there was an opposite situation in the pedestrian group. Considering the new design tends to give priority to pedestrians as “vulnerable groups”, the restriction to motor vehicles has increased.

#### Interrelationship among different criterias and the influence of aesthetic elements

The results of Spearman’s test presents that improving one criteria is likely to improve street users’ evaluation of other criterias. For example, in pedestrian group, scenario C with the highest safety MPR score also has a relatively higher performance in walking experience, which corresponds to the correlation between “safety” and “walking experience” criteria in the Spearman matrix. As proposed in much literature, shared streets can effectively ensure traffic safety [5, 40]. The reason is that this kind of street layout can improve the comfort of users while removing the hard isolation, improving the traffic environment and creating mental speed bump for users. By exploiting the correlation among different criterias of street performance, speeding caution can be gave to both drivers and pedestrians.

Moreover, aesthetic elements is a significant factor to affect performance rank considering its importance in improving comfort, walking/ driving experience and environment. For example, in scenario B, the change of pavement types, curved street design, and the location of landscape help drivers to drive carefully while increasing the sense of pleasure. Concerning the higher MPR value of safety, priority, and environmental criteria in both groups, it can be confirmed that aesthetic elements do have positive effects on other criterias in this experimental environment. Especially the participates in the driver group gave a strongly positive attitude.

#### Limitations

There are some limitations related to participates. The limitation is that there were participants who had discomfort wearing VR equipment (12.50% for the pedestrian group and 18.75% for the driver group, respectively). Since this research does not consider the dizziness during the experiment, the more sample is required to get a less negative effect on the result from the discomfort. Besides, due to the limitation of COVID-19, the sample size cannot be fully guaranteed, which may have a certain impact on the accuracy of the experimental results. Taking into account the adaptability of college students to the new VR technology, the selected participants are generally young. Because the street, as an inclusive public space, should meet the needs of all users, participants of different age groups should be considered in further study. With respect to quantitative analysis of safety performance, although the results show that people were optimistic that shared streets could improve safety, there was a lack of systematic safety quantitative analysis. For further research, it could be much useful if more traffic simulation software can be added and conduct a safety analysis from a micro perspective.

## CONCLUSION

This study aims at exploring the applicability and recognition of shared streets in China from both pedestrians and drivers’ perceptions. Taken an 800 meters long street in front of Suzhou Museum as a case, Virtual Reality and HITL technology are utilized in this research. This study develops three street layouts that incorporate the Chinese environment, in which the road facilities and landscape layout adopt Chinese style. By making some improvements and adjustments to this system and adding more software collaborations, the entire virtual environment can be highly visualized. With the simulation experiment from both driver and pedestrian perception, responses regarding the opinion of shared streets and design preferences are collected for the following analysis.

The separation of sidewalks and carriageway is the fundamental difference between the three scenarios. Scenario A removes all delimitations, scenario C has relatively high separation of people and vehicles, and scenario B has both A and C characteristics. Although the perception of this design varies significantly due to individual differences, high recognition represented in all the three scenarios in terms of amenity and safety criteria. The comprehensive evaluation shows that the participates in the pedestrian group gave the similar scores for scenarios B and C, and are much higher than A. In the other experimental group, the driver preferred scenario C and gave the lowest score for scenario B. This result present that pedestrians have a high degree of recognition for the design of people-car sharing while drivers have reservations about the design that create more uncertainties for the driving process. Combining the perceptions of the two groups of participates, the scenario C with the maximum soft isolation can get the relative highest support in this condition.

Furthermore, there is complex interrelationship existed among different criterias. The results reveal that aesthetic street furniture is also a decisive element to enhance safety and improve user satisfaction when using the street. The rational setting of the ground frontage and the spatial treatment of paving can also have a positive impact on improving street safety and stimulating vitality. The reasonable combination of vertical aesthetics and environmental utilization can increase the uncertainty of the street environment visually and sensory, thereby enhancing the vigilance of street users.

Lastly, for future work, more type of streets users should be considered in experiment to develop the potential of shared streets. The street facilities that can provide convenience for the elderly, children and the disabled can be added streets design and test using this analysis framework in further study.

## ACKNOWLEDGMENTS

This research was funded by Key Project of Philosophy and Social Science Research in Colleges and Universities in Jiangsu Province (No. 2020SJZDA133).

## REFERENCE

[1] Karndacharuk, A., D.J. Wilson, and R. Dunn, *A review of the evolution of shared (street) space concepts in urban environments.* Transport reviews, 2014. **34**(2): p. 190-220.

[2] Gilman, C., R. Gilman, and M.P. Tem. *Shared-Use Streets–An Application of “Shared Space” to an American Small Town*. in *3rd Urban Street Symposium, Seattle, WA, USA*. 2007.

[3] Jayakody, R.R., et al., *Design Factors for a Successful Shared Street Space (SSS) Design.* International Journal of Strategic Property Management, 2018. **22**(4): p. 278-289.

[4] Biddulph, M., *Home Zones: A planning and design handbook*. 2001: Policy Press.

[5] Hamilton-Baillie, B., *Shared space: Reconciling people, places and traffic.* Built environment, 2008. **34**(2): p. 161-181.

[6] Biddulph, M., *Towards successful home zones in the UK.* Journal of Urban Design, 2003. **8**(3): p. 217-241.

[7] Hamilton-Baillie, B., *Towards shared space.* Urban Design International, 2008. **13**(2): p. 130-138.

[8] Engwicht, D., *Mental Speed Bumps: The smarter way to tame traffic*. 2005: Envirobook.

[9] Ruiz-Apilánez, B., et al., *Shared space streets: design, user perception and performance.* Urban Design International, 2017. **22**(3): p. 267-284.

[10] Obeid, H., et al., *Analyzing driver-pedestrian interaction in a mixed-street environment using a driving simulator.* Accident Analysis & Prevention, 2017. **108**: p. 56-65.

[11] Charlton, S.G., et al., *Using endemic road features to create self-explaining roads and reduce vehicle speeds.* Accident Analysis & Prevention, 2010. **42**(6): p. 1989-1998.

[12] Karndacharuk, A., *The Development of a multi-faceted evaluation framework of shared spaces*. 2014, ResearchSpace@ Auckland.

[13] Jayaram, S., H.I. Connacher, and K.W. Lyons, *Virtual assembly using virtual reality techniques.* Computer-aided design, 1997. **29**(8): p. 575-584.

[14] Lok, B., et al., *Applying virtual reality in medical communication education: current findings and potential teaching and learning benefits of immersive virtual patients.* Virtual Reality, 2006. **10**(3-4): p. 185-195.

[15] Tsai, M.-D., M.-S. Hsieh, and S.-B. Jou, *Virtual reality orthopedic surgery simulator.* Computers in biology and medicine, 2001. **31**(5): p. 333-351.

[16] Meggs, S.M., A. Greer, and S. Collins, *Virtual reality in interior design education: Enhanced outcomes through constructivist engagement in Second Life.* International Journal of Web-Based Learning and Teaching Technologies (IJWLTT), 2012. **7**(1): p. 19-35.

[17] Portman, M.E., A. Natapov, and D. Fisher-Gewirtzman, *To go where no man has gone before: Virtual reality in architecture, landscape architecture and environmental planning.* Computers, Environment and Urban Systems, 2015. **54**: p. 376-384.

[18] Anthes, C., et al. *State of the art of virtual reality technology*. in *2016 IEEE Aerospace Conference*. 2016. IEEE.

[19] Mihelj, M., D. Novak, and S. Beguš, *Virtual reality technology and applications.* 2014.

[20] Velev, D. and P. Zlateva, *Virtual reality challenges in education and training.* International Journal of Learning and Teaching, 2017. **3**(1): p. 33-37.

[21] Meir, A., T. Oron-Gilad, and Y. Parmet, *Are child-pedestrians able to identify hazardous traffic situations? Measuring their abilities in a virtual reality environment.* Safety science, 2015. **80**: p. 33-40.

[22] Chun, W., et al. *Virtual-reality based integrated traffic simulation for urban planning*. in *2008 International Conference on Computer Science and Software Engineering*. 2008. IEEE.

[23] Sütfeld, L.R., et al., *How does the method change what we measure? Comparing virtual reality and text-based surveys for the assessment of moral decisions in traffic dilemmas.* PloS one, 2019. **14**(10): p. e0223108.

[24] Feng, D., et al. *Learning Generative Models of Social Interactions with Humans-in-the-Loop*. in *2018 17th IEEE International Conference on Machine Learning and Applications (ICMLA)*. 2018. IEEE.

[25] Kefalidou, G., et al. *Designing Airport Interiors with 3D Visualizations*. in *Extended Abstracts of the 2019 CHI Conference on Human Factors in Computing Systems*. 2019.

[26] Jiang, L., et al., *How do shared-street design and traffic restriction improve urban soundscape and human experience?—An online survey with virtual reality.* Building and Environment, 2018. **143**: p. 318-328.

[27] Deb, S., et al., *Efficacy of virtual reality in pedestrian safety research.* Applied ergonomics, 2017. **65**: p. 449-460.

[28] Lei, W., et al. *Research on hybrid electrical vehicle based on human-in-the-loop simulation*. in *2014 IEEE Conference and Expo Transportation Electrification Asia-Pacific (ITEC Asia-Pacific)*. 2014. IEEE.

[29] Hartmann, M., et al. *“Pedestrian in the Loop”: An approach using virtual reality*. in *2017 XXVI International Conference on Information, Communication and Automation Technologies (ICAT)*. 2017. IEEE.

[30] Oh, T. *The Use of Emerging Virtual Reality Technology in Road Safety Analysis : The Hook-Turn Case*. 2019.

[31] Biddulph, M., *Radical streets? The impact of innovative street designs on liveability and activity in residential areas.* Urban Design International, 2012. **17**(3): p. 178-205.

[32] Manzo, L., *On people in changing neighborhoods. Gentrification and social mix: boundaries and resistance. A comparative ethnography of two historic neighborhoods in Milan (Italy) and Brooklyn (New York, USA).* CIDADES: Comunidades e Territórios, Portuguese, 2012. **24**: p. 1-29.

[33] Mackie, H.W., et al., *Road user behaviour changes following a self-explaining roads intervention.* Accident Analysis & Prevention, 2013. **50**: p. 742-750.

[34] Sauter, D. and M. Huettenmoser, *Liveable streets and social inclusion.* Urban Design International, 2008. **13**(2): p. 67-79.

[35] Allen, D. *PERS v2: Auditing public spaces and interchange spaces. Walk21-VI" Everyday Walking Culture*. in *The 6th International Conference on Walking in the 21st Century*. 2005.

[36] Clifton, K.J., A.D.L. Smith, and D. Rodriguez, *The development and testing of an audit for the pedestrian environment.* Landscape and urban planning, 2007. **80**(1-2): p. 95-110.

[37] Bradburn, N.M., S. Sudman, and B. Wansink, *Asking questions: the definitive guide to questionnaire design--for market research, political polls, and social and health questionnaires*. 2004: John Wiley & Sons.

[38] Torgerson, W.S., *Theory and methods of scaling.* 1958.

[39] Karndacharuk, A., P. Vasisht, and M. Prasad. *Shared Space Evaluation: O’Connell Street, Auckland*. in *Proceedings of the Australasian Transport Research Forum*. 2015.

[40] Quimby, A. and J.A. Castle, *A review of simplified streetscape schemes*. 2006: TRL Limited London.

1. Withdrawal: If you agree to participate in this study, you will need to agree to these terms at the start of the survey. And you can withdraw any stage of the investigation at any time during the process.

   Confidentiality: Only the thoughts and opinions volunteered by the participants in the survey will identified in the data collection, and none of them will be identified by name. The findings will be used for publication purposes in the form of papers, journal articles or conferences. [↑](#footnote-ref-1)
