# Supplementary material for: Are shared streets acceptable to pedestrians and drivers? Evidence from Virtual Reality experiments
Source: PLoS One. 2022 Apr 15;17(4):e0266591. doi: 10.1371/journal.pone.0266591 (PMC9012376; doi:10.1371/journal.pone.0266591)
Supplement: S1 File — (ZIP) [file pone.0266591.s001.zip › supprting information/questionnaire/questionnaire-driver sheet.pdf]

The Applicability of Shared Streets in China with Virtual Reality
(Driver Group Sheet)

Please circle the option best describes your opinion towards the following five statements. (-3 to 3 represent Strongly Disagree, Disagree, Tend to Disagree, Tend to Agree, Agree, Strongly Agree, respectively.)

1) "I felt comfortable driving"	-3	-2	-1	1	2	3
2) "I could drive smoothly"	-3	-2	-1	1	2	3
3) "I would like to drive slowly"	-3	-2	-1	1	2	3
4) "I preferred to yield to pedestrians"	-3	-2	-1	1	2	3
5) "I felt the traffic noise is reduced compared to conventional streets"	-3	-2	-1	1	2	3

Please further provide us with your impression about the street space and your background information

6) Have you ever heard of the shared street before? (a)Yes, (b)No.
7) Have you ever experienced VR before? (a)Yes, (b)No.
8) Do you ever feel dizzy using the VR simulator? (a)Yes, (b)No.
9) Your opinion of shared street's layout compared to conventional streets?

____

10) Please fill in your age.

____

11) Gender: ☐ Male ☐ Female
