# Supplementary material for: Are shared streets acceptable to pedestrians and drivers? Evidence from Virtual Reality experiments
Source: PLoS One. 2022 Apr 15;17(4):e0266591. doi: 10.1371/journal.pone.0266591 (PMC9012376; doi:10.1371/journal.pone.0266591)
Supplement: S1 File — (ZIP) [file pone.0266591.s001.zip › supprting information/questionnaire/questionnaire-Participant Information Sheet.pdf]

Participant Information Sheet

The Applicability of Shared Streets in China with Virtual Reality

Dear Participant:

Researcher Introduction

My name is Lurong Xu. I am a master student of Southeast University. My supervisor is Dr. Xiaojian Hu in the Department of Transportation Engineering. Taeho OH, a joint PHD student from Southeast University and Monash University supervised by Dr. Inhi Kim in the Department of Civil Engineering from Monash University will be assisting me in the survey process.

Project Description and Invitation

You are warmly invited to participate in our research, and I would appreciate any assistance you can offer. You have been identified as a road user equipped with Virtual Reality tools to experience virtual shared streets. We are exploring the applicability of the conception of shared streets in China and how road spaces designed as a shared space perform their 'movement, access and place' functions. This research aims to develop a framework in evaluating the performance of shared space schemes, taking into account both qualitative and quantitative performance data.

Project Procedures

We would like to invite you to engage in virtual shared streets experiment developed by VR technology to share your opinions and experience, and to explore how you perceive the effectiveness of the different layouts of the three virtual shared streets scenarios. The experiment will take approximately 15 minutes and up to 30 minutes.

Since the performance of a shared space can be evaluated by considering how well a street contributes towards fulfilling the shared space objectives, interview questions for each study area will be related to the aim of placemaking, pedestrian focus, vehicle behavior change, economic impetus and safety for all users. The interview questions are attached.

Right to Withdraw from Participation

You have the right to withdraw from participation at any time. You may withdraw your data from the study at any time for up to one month following the interview.

Data Collection, and Future Use

The data for this study will be collected and stored as paper documents. The responses from this

questionnaire may be used in other studies such as PhD or master research projects and may be used for publication purposes.

Confidentiality of Responses

We will keep all responses during the interview session completely confidential. Responses will be kept in a locked file to which only the researchers and supervisor have access. It cannot be completely guaranteed that participant anonymity will be preserved, however the researchers will strive to protect the anonymity of participants at all stages of the research. Individuals' names and other identifying information will be disguised in the research report. The researchers will report what is said, but will not attribute statements to identifiable individuals. All participants, and their employers, will be given the opportunity to review the final report before it is published externally.

Contact Details

Thank you very much for your time and help in making this study possible. If you wish to know more about the study, or have any concerns, please email, phone, or write to me at:

Lurong Xu
Jiangsu Key Laboratory of Urban ITS， Southeast University;
Jiangsu Province Collaborative Innovation Center of Modern Urban Traffic Technologies, Southeast University;
Monash Institute of Transport Studies, Department of Civil Engineering,
Monash University, Clayton, Victoria 3800, Australia.
Email: 782145216@qq.com

My supervisor is:

Xiaojian Hu (Corresponding Author)
Ph.D., Associate Professor
Jiangsu Key Laboratory of Urban ITS， Southeast University；
Jiangsu Province Collaborative Innovation Center of Modern Urban Traffic Technologies, Southeast University；
School of Transportation, Southeast University
Southeast University Road #2, Nanjing, 211189, China
Email: huxiaojian@seu.edu.cn

APPROVED BY THE SOUTHEAST UNIVERSITY HUMAN RESEARCH ETHICS COMMITTEE on 01/04/2020 until 01/04/2025
